# Mechanical unfolding kinetics of the SRV-1 gag-pro mRNA pseudoknot: possible implications for −1 ribosomal frameshifting stimulation

**DOI:** 10.1038/srep39549

**Published:** 2016-12-21

**Authors:** Zhensheng Zhong, Lixia Yang, Haiping Zhang, Jiahao Shi, J. Jeya Vandana, Do Thuy Uyen Ha Lam, René C. L. Olsthoorn, Lanyuan Lu, Gang Chen

**Affiliations:** 1 Division of Chemistry and Biological Chemistry, School of Physical and Mathematical Sciences, Nanyang Technological University, 21 Nanyang Link, 637371 Singapore; 2 School of Biological Sciences, Nanyang Technological University, 60 Nanyang Drive, 637551 Singapore; 3 St Andrew’s Junior College, 5 Sorby Adams Drive, 357691 Singapore; 4 Leiden Institute of Chemistry, Leiden University, P.O. Box 9502, 2300 RA Leiden, The Netherlands

## Abstract

Minus-one ribosomal frameshifting is a translational recoding mechanism widely utilized by many RNA viruses to generate accurate ratios of structural and catalytic proteins. An RNA pseudoknot structure located in the overlapping region of the *gag* and *pro* genes of Simian Retrovirus type 1 (SRV-1) stimulates frameshifting. However, the experimental characterization of SRV-1 pseudoknot (un)folding dynamics and the effect of the base triple formation is lacking. Here, we report the results of our single-molecule nanomanipulation using optical tweezers and theoretical simulation by steered molecular dynamics. Our results directly reveal that the energetic coupling between loop 2 and stem 1 via minor-groove base triple formation enhances the mechanical stability. The terminal base pair in stem 1 (directly in contact with a translating ribosome at the slippery site) also affects the mechanical stability of the pseudoknot. The −1 frameshifting efficiency is positively correlated with the cooperative one-step unfolding force and inversely correlated with the one-step mechanical unfolding rate at zero force. A significantly improved correlation was observed between −1 frameshifting efficiency and unfolding rate at forces of 15–35 pN, consistent with the fact that the ribosome is a force-generating molecular motor with helicase activity. No correlation was observed between thermal stability and −1 frameshifting efficiency.

Recent advances in the discovery and understanding of complex RNA structures, dynamics, and functions have dramatically changed our view of RNA’s role in biology. RNA has been found to play increasingly important and diverse regulatory and catalytic roles in many biological processes ranging from replication, transcription, splicing, to translation^1^. In this study, we focus on understanding how the translation reading frame can be regulated by *cis*-acting mRNA structures. The translation process is highly regulated and remarkably accurate. Normally, the reading frame needs to be maintained at a constant frame (0 frame) because shifting of the reading frame results in the generation of completely different protein products. Minus-one ribosomal frameshifting is a translational regulatory mechanism programmed by *cis*-acting mRNA structures and widely utilized by viruses and cells^2^,^3^,^4^,^5^,^6^,^7^,^8^,^9^,^10^,^11^,^12^,^13^. During −1 ribosomal frameshifting, a ribosome is positioned at a certain sequence of mRNA called slippery sequence, and has a certain probability to move one nucleotide backward, before continuing translation in the new reading frame (−1 frame)^11^,^14^,^15^. Minus-one frameshifting is important for (**i**) maintaining a fixed ratio of certain gene products through the translation of a fusion protein by bypassing the original 0 frame stop codon, and (**ii**) regulating the half-lives of mRNAs by employing mRNA degradation pathways through premature translation termination at a −1 frame stop codon^11^,^13^.

Remarkably, the shifting of the mRNA reading frame of a megadalton ribosome complex can be manipulated by *cis*-acting mRNA sequences and structures. The slippery mRNA sequence in viruses and eukaryotes is usually X XXY YYZ (with the underlined codons specifying the 0 frame), where XXX are any 3 identical nucleotides, YYY are either AAA or UUU, and Z is usually A, C or U. An mRNA structure located downstream of the slippery sequence separated by a single-stranded spacer (2 to 8 nucleotides (nt) in length) stimulates −1 frameshifting from 0 frame X XXY YYZ to −1 frame XXX YYY Z. The downstream frameshifting stimulatory structure can be a hairpin (stem-loop)^6^,^16^,^17^, a pseudoknot^5^,^8^,^9^,^10^,^12^,^15^,^18^,^19^, a duplex formed between the mRNA and an antisense oligonucleotide^20^,^21^,^22^,^23^, or a G-quadruplex^24^,^25^.

In addition to the downstream mRNA structure, the slippery sequence pattern and spacer length can also significantly affect the −1 frameshifting efficiency^14^,^26^,^27^,^28^. Chemical mapping (Footprinting) studies^29^ of beet western yellow virus (BWYV) pseudoknot-*E coli* ribosome complexes show that the first base pair in stem 1 of a pseudoknot may melt in the presence of a ribosome at the slippery site if the single-stranded spacer is shortened from 6 to 5 nt. Clearly, slippery sequence, single-stranded spacer length, and downstream structure are strongly coupled in determining the −1 ribosomal frameshifting.

Significant advances have been recently made in understanding the conformational dynamics and kinetic partitioning of the prokaryotic ribosome complex, and (un)binding dynamics of tRNA and elongation factor G (EF-G) during the −1 frameshifting process^30^,^31^,^32^,^33^,^34^. A mechanistic picture emerging from the conformational and compositional dynamics studies is that a downstream mRNA pseudoknot or hairpin structure may trap the ribosome at a rotated pre-translocation conformation and slow down the subsequent translation steps in the 0 frame. Upon unfolding the downstream mRNA structure by the ribosome, the ribosome rotates back to the normal conformation to release the tension. In the process of unfolding of the downstream structure, the ribosome has a probability to slip into an alternative frame at the slippery site, eventually resulting in the accommodation of a new cognate aminoacyl-tRNA on the first codon in the −1 frame.

It is critical to understand how the mechanical properties of the downstream *cis*-acting mRNA structure affect −1 frameshifting efficiency. However, the molecular determinants of the downstream mRNA structure stimulating −1 frameshifting efficiency are still unclear. Single-molecule mechanical pulling studies using optical tweezers have provided insight into the possible correlation between frameshifting stimulatory mRNA structures and −1 frameshifting efficiency^35^,^36^,^37^,^38^,^39^,^40^. Two studies showed a correlation between the frameshifting efficiency and the mechanical strength of an mRNA pseudoknot^35^,^37^. Both major-groove and minor-groove base triples in an mRNA pseudoknot were found to increase mechanical stability and −1 frameshifting efficiency^37^. Green *et al*. suggested that a slower unfolding rate correlates with higher −1 frameshifting efficiency over a narrow force range^36^. However, Ritchie *et al*. proposed a mechanism in which the frequency of the formation of alternative mRNA structures is proportional to −1 frameshifting efficiency^38^.

A hairpin-type (H-type) pseudoknot structure (Fig. 1) is formed in the overlapping region of the *gag* and *pro* genes of Simian retrovirus type 1 (SRV-1). An NMR study revealed that minor-groove base triples form between stem 1 and loop 2 of the pseudoknot^18^. Extensive mutagenesis assays have revealed that the frameshifting efficiencies are highly dependent on the loop residues in the pseudoknot^18^,^19^. A coarse-grained simulation suggests a cooperative thermal unfolding of the two stems of the SRV-1 pseudoknot^41^. However, the experimental characterization of SRV-1 pseudoknot stability and (un)folding dynamics and the effect of the base triple formation is lacking. Here we combined mechanical and thermal unfolding, electrophoretic mobility assays, and Molecular Dynamics (MD) simulation studies of SRV-1 RNA frameshifting pseudoknots (Fig. 1 and Supplementary Fig. S1) to unravel the molecular determinants of an mRNA pseudoknot in stimulating frameshifting.

## Results and Discussion

**Figure 1:**
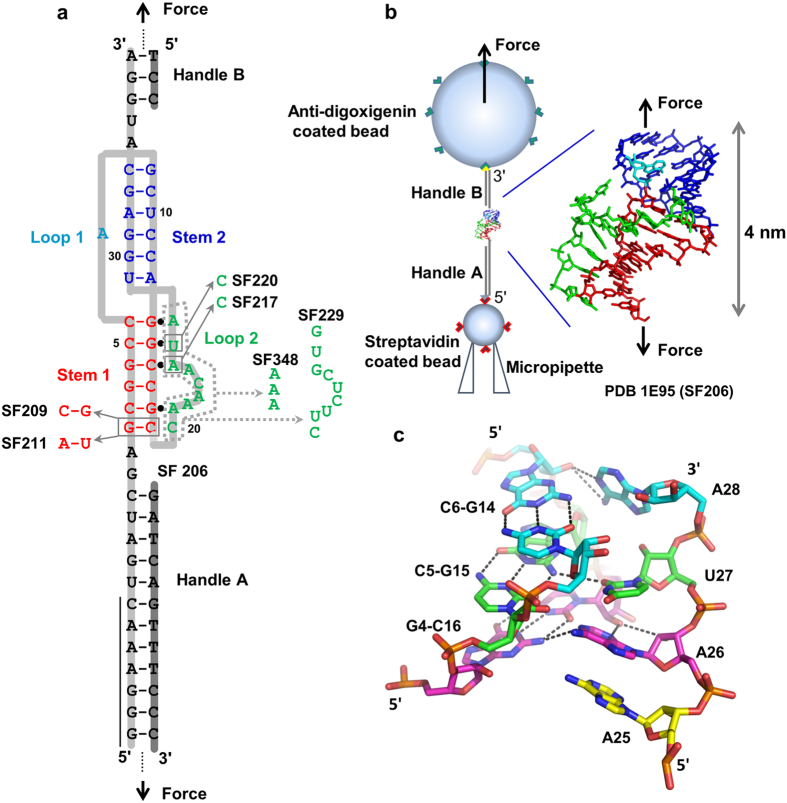
Pseudoknot structures and optical tweezers experimental setup. **(a**) Secondary structure of pseudoknot SF206 and mutants. The slippery sequence (G GGA AAC) is indicated by a vertical black line. The DNA strands (highlighted in dark gray) are complementary to the mRNA (highlighted in light gray) forming the handles A and B for the pulling experiments. (**b**) Experimental setup of single-molecule force spectroscopy using Minitweezers. The NMR structure^18^ is shown with the same color coding as panel **a**. The drawing is not to scale. (**c**) Structures of the three consecutive minor-groove base triples A28∙C6-G14 (cyan), U27∙C5-G15 (green), and A26∙G4-C16 (magenta) found in SF206.

Previously, an *in vitro* frameshifting assay was performed with all constructs containing the same slippery sequence (G GGA AAC) and a 7-nt single-stranded spacer^19^. A 7-nt spacer (UGAUCGA) is present in SF206, SF209 and SF211, and a slightly different spacer (UAGUUGA) is present in the four remaining constructs. Neither of the two spacer sequences is expected to affect the (un)folding dynamics of the downstream pseudoknot which has two G-C pair-rich stems (Fig. 1a). The mRNA-ribosome interaction is assumed not to be significantly affected by the small change of the unstructured single-stranded spacer. The frameshifting pseudoknots chosen in our studies include the “wild-type” pseudoknot (SF206) with the three-dimensional structure revealed by NMR^18^, and a series of mutants (see Fig. 1a and Supplementary Fig. S1) whose design was based on the NMR structure^19^. The structure of SF206 reveals consecutive minor-groove base triples formed between stem 1 and loop 2 (Fig. 1c). Mutations were designed to disrupt the base triples and the base stacking interactions expected to affect frameshifting efficiencies^19^. The previous mutational studies^19^ revealed that SF206 and the six mutants display *in vitro* frameshifting efficiencies in the range between 21% and 1%, which allows us to investigate the possible coupling effect between stem 1 and loop 2 on thermal and mechanical stabilities of the frameshifting pseudoknots, and the possible correlation with the frameshifting efficiency.

### mRNA thermal stability has no correlation with *in vitro* frameshifting efficiency

**Figure 2:**
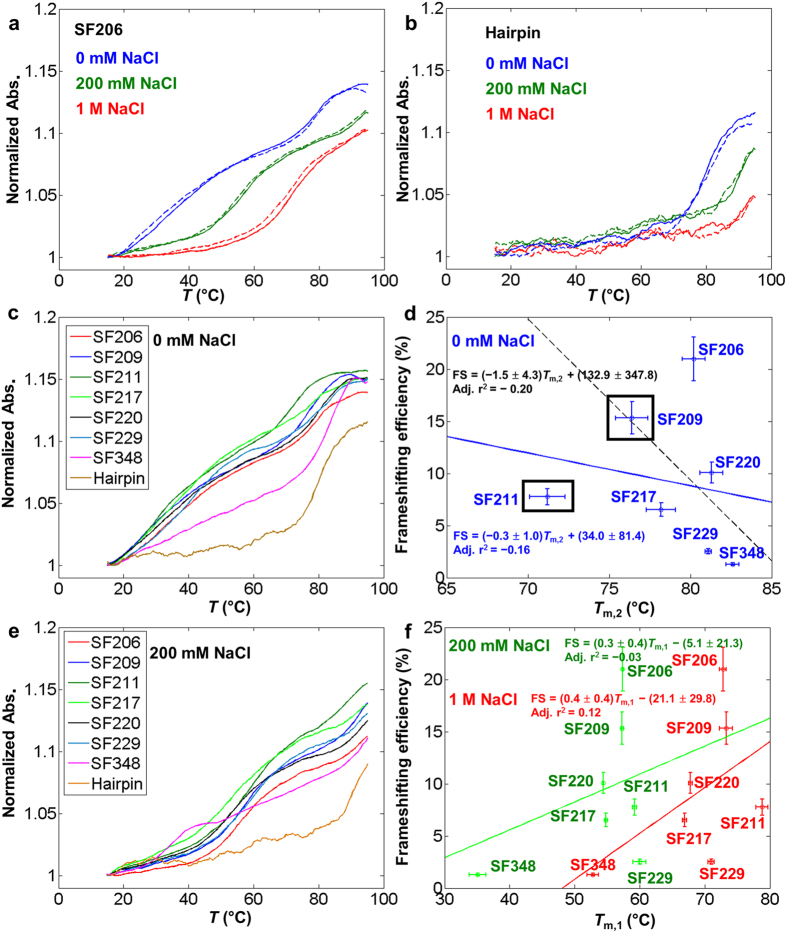
Ensemble thermal melting studies reveal no correlation between thermal stability and frameshifting efficiency. Error bars for *T*_m,1_, *T*_m,2_ and frameshifting efficiency represent standard deviations. (**a**) Normalized UV melting trajectories of pseudoknot SF206 in the buffers with 1 M (red), 200 mM (green) and 0 mM (blue) NaCl, respectively. Solid lines represent the heating curve and dashed lines represent the cooling curve. (**b**) Normalized UV melting trajectories of isolated stem 1 hairpin (containing residues from 1–19) in the buffers with 1 M (red), 200 mM (green) and 0 mM (blue) NaCl, respectively. Solid lines represent the heating curve and dashed lines represent the cooling curve. (**c**) Normalized UV melting trajectories of pseudoknots and control hairpin in the buffer with 0 mM NaCl. SF348 shows only the melting of stem 1 suggesting that the pseudoknot structure for SF348 does not form at this condition. (**d**) No correlation is observed between *T*_m,2_ in 0 mM NaCl and *in vitro* frameshifting efficiency previously measured^19^. The blue solid line and the black dashed lines represent the linear fitting curves for data points with and without SF209 and SF211 included, respectively. (**e**) Normalized UV melting trajectories of pseudoknots and control hairpin in the buffer with 200 mM NaCl. (**f**) No correlation is observed between *T*_m,1_ value in 200 mM NaCl (green) and 1 M NaCl (red) and previously measured *in vitro* frameshifting efficiency^19^. The green and red lines represent the linear fitting curves for thermal melting data points obtained at 200 mM and 1 M NaCl, respectively.

The NMR structure for the “wild-type” SRV-1 pseudoknot SF206 (containing the residues 1–34, see Fig. 1b) was solved in a buffer containing 100 mM NaCl, 10 mM KHPO_4_/KH_2_PO_4_ (pH 6.4)^18^. We measured the ensemble thermal unfolding of the oligonucleotide constructs for the pseudoknots (containing the residues 1–34) and a control hairpin (containing the residues 1–19) in 20 mM HEPES, 0.1 mM EDTA, pH 7.3 with varying NaCl concentrations (0 mM, 200 mM or 1 M NaCl). In the thermal (un)folding experiments, all the melting curves are superimposable for heating and cooling curves (Fig. 2a,b), suggesting the thermal unfolding and folding transitions are reversible. In the buffer with 0 mM NaCl, except for SF348, two apparent transitions (with melting temperatures at *T*_m,1_ and *T*_m,2_, *T*_m,1_ < *T*_m,2,_) are observed for all pseudoknots (Fig. 2a,c). In 0 mM NaCl, the *T*_m,2_ of SF206 (~80 °C, Fig. 2a) is similar to the melting temperature of the control hairpin (stem 1 hairpin) (Fig. 2b), suggesting that stem 2 (*T*_m,1_) melts before stem 1 (*T*_m,2_) in the SRV pseudoknots. SF348 shows two-step thermal melting in the buffers containing 200 mM (Fig. 2e) and 1 M NaCl (Data not shown), suggesting that stem 2 in SF348 is formed in 200 mM and 1 M NaCl but not in 0 mM NaCl. SF348 has a significantly lower *T*_m,1_ compared to other pseudoknots, probably because the shortened loop 2 creates steric strain resulting in partial deformation of stem 2 and/or unstacking with stem 1.

According to the nearest-neighbour thermodynamic model^42^,^43^,^44^, changing a G-C (SF206) to a C-G (SF209) and an A-U (SF211) pair (Fig. 1a) destabilize stem 1 by 0.5 or 1.9 kcal/mol (penalty for terminal A-U pair is considered), respectively, at 1 M NaCl and 310 K (predicted using RNAstructure 5.3). Consistently, in 0 mM NaCl, the *T*_m,2_ values of SF209 (76.4 °C) and SF211 (71.2 °C) are significantly lower than those of other pseudoknots (Fig. 2c,d). As expected, except for SF209 and SF211, the *T*_m,2_ of the pseudoknots in 0 mM NaCl are in a narrow range between 78 and 82 °C, which is close to the melting temperature of control stem 1 hairpin (80.1 °C). The SRV pseudoknots and the control stem 1 hairpin do not completely melt even at 95 °C (Fig. 2a,b,e) in the buffer with 200 mM or 1 M NaCl. Taken together, our thermal melting profiles suggest that the transition with higher melting temperatures (*T*_m,2_) corresponds to the thermal unfolding of stem 1 in the pseudoknots. The two-step melting pathway observed in our study is in agreement with the coarse-grained simulation for SF206, although an apparent one-step melting transition was observed in the simulation^41^.

Stem 1 is directly in contact with a ribosome during the −1 frameshifting process, and its stability may correlate with frameshifting efficiency. However, our thermal melting data show no obvious correlation between *T*_m,2_ and *in vitro* frameshifting efficiency measured previously^19^ by using a commercially available *in vitro* translation system (reticulocyte lysate (Promega)) (Fig. 2d). The absence of a correlation is consistent with previous observations^37^ and is likely due to the fact that the formation of stem 2 and minor-groove triples (formed between stem 1 and loop 2) in a pseudoknot may exert a coupling effect in stabilizing stem 1 as has been observed in recent nanopore pseudoknot unzipping experiments^45^. The thermodynamic stabilization effect due to the coaxial stacking between stem 1 and stem 2 and stem 1-loop 2 bases triple formation is not observed in the thermal denaturation, because under the global temperature perturbation, the base triples and stem 2 melt before stem 1.

Previous NMR and mutational studies revealed that a triplex structure is formed between stem 1 and loop 2 and disruption of the base triples decreases the frameshifting efficiency^18^,^19^. Thus, the triplex stability may correlate with the frameshifting efficiency. However, the thermal melting transitions for the triplex and stem 2 seem not to be resolved, resulting in one overlapped transition (Fig. 2a,c,e). Thus, the low-temperature transition (*T*_m,1_, Fig. 2a,c,e) of the pseudoknots corresponds to the melting of both the tertiary triplex structure and stem 2. We did not observe a clear correlation between *T*_m,1_ (in 200 mM or 1 M NaCl) and previously measured *in vitro* frameshifting efficiency (Fig. 2f)^19^. The absence of a correlation might be due to the fact that the melting of the triplex (formed between stem 1 and loop 2) and duplex (stem 2) are not coupled, *i.e.*, triplex and stem 2 melt independently, but with an overlapped broad melting transition. The relatively broad melting transitions and limited resolution of the experimental data prevented us in extracting the thermodynamic parameters for the transitions^9^,^15^,^46^. Taken together, our experimental ensemble thermal melting results reveal no correlation between thermal stability and *in vitro* frameshifting efficiency.

### Replica-Exchange Molecular Dynamics (REMD) simulation provides insights into atomistic thermal unfolding mechanism

We carried out REMD simulation for SF206 to reveal the detailed mechanism of the thermal unfolding process. We monitored the thermal unfolding process based on the average numbers of the hydrogen bonds formed in stem 1, stem 2 and stem 1-loop 2 triplex, respectively, in each replica of the REMD simulation (Supplementary Fig. S2). We observed that both stem 2 and stem 1-loop 2 triplex start to thermally melt at 351 K, with half of the hydrogen bonds disrupted between 449 and 461 K. Stem 1 starts to melt at around 390 K, with half of hydrogen bonds melted between 461 and 481 K. Furthermore, the detailed thermal unfolding pathway and intermediate structures can be revealed by the two-dimensional free energy surface plots (Supplementary Fig. S3). Taken together, the REMD simulation data suggest that stem 2 and stem 1-loop 2 triplex thermally unfold before stem 1, which is consistent with the unfolding pathway suggested by the experimental thermal melting data.

The simulated melting temperature ranges (449–461 K for stem 2 and stem 1-loop 2 triplex and 461–481 K for stem 1) are higher than the experimental melting temperatures. A relatively high simulated melting temperature was also observed in the MD simulation of another RNA pseudoknot^47^. Thus, the discrepancy in temperature values might be due to the limitation of the force field and/or relatively short simulation time. Nevertheless, our simulation results confirm the relative stem stability observed in the thermal melting experiments and provide insights into the atomistic mechanism of the thermal unfolding of the SRV-1 pseudoknot. Both experimental thermal melting data and the REMD simulation data suggest that stem 2 and stem 1-loop 2 triplex thermally unfold before stem 1 in SF206.

### Base triple formation makes the mRNA pseudoknot more compact

**Figure 3:**
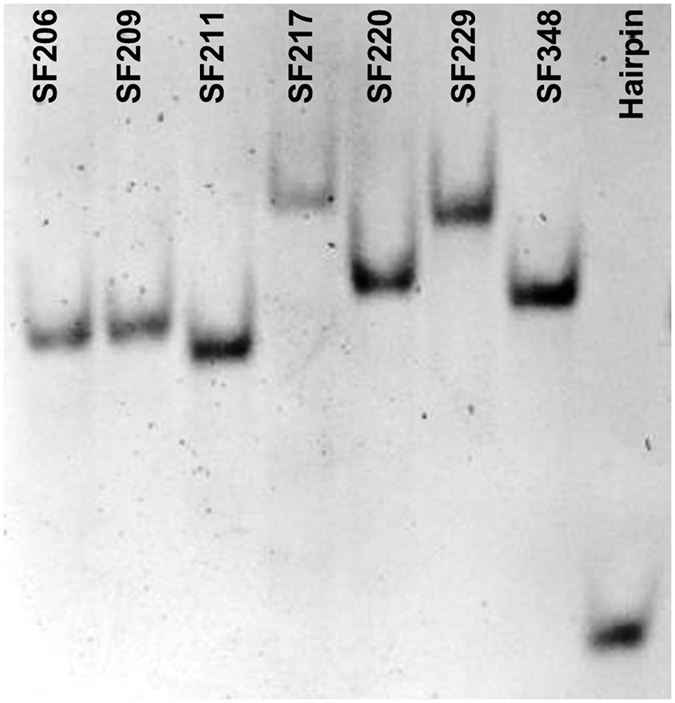
Native PAGE results for RNA oligonucleotides (consisting of residues from 1 to 34 for the pseudoknots and residues 1–19 for the hairpin containing stem 1). The same constructs were used for the thermal melting studies. The stem 1-loop 2 minor-groove base triple formation is maintained in SF206, SF209, and SF211. The mutants SF217 and SF220 have a single base mutation of a residue in loop 2 involved in base triple interactions.

We carried out a native polyacrylamide gel electrophoresis (PAGE) experiment with the RNA oligonucleotide constructs (residues 1–34 for the pseudoknots and 1–19 for the hairpin containing stem 1) to reveal if the structural compactness correlates with the base triple formation (Fig. 3). We used a loading (incubation) buffer containing 200 mM NaCl, as our thermal melting experiments reveal that all the SRV-1 pseudoknot constructs can fold into the native structures in 200 mM NaCl (Fig. 2e). The migration rate of the stem 1 hairpin is significantly faster than the pseudoknots, which is consistent with the fact that stem 1 hairpin is smaller than the pseudoknots. The migration rates of SF206, SF209 and SF211 are similar, presumably due to the fact that the loop 2 sequences of these three pseudoknots are the same with the stem 1-loop 2 minor-groove triplex structure forming.

It is remarkable that SF217 (with a simple A26C mutation in loop 2) and SF229 (with a relatively bigger change in loop 2) show the slowest migration, presumably because the stem 1-loop 2 base triple structures are disrupted in both mutants. The data suggest the critical role of A26 in stabilizing the stem 1-loop 2 triplex and compacting the pseudoknot structure. A single hydrogen bond is formed between U27 and G15 in the U27∙G15-C5 base triple (Fig. 1a,c)^18^, and the U27C mutation in SF220 is not expected to disrupt the hydrogen bond. However, SF220 has a slightly slower migration compared to SF206, which may be due to an unfavorable stacking between A26 and C27 (both with an exocyclic amine group) resulting in a loosened loop 2. SF348 has a similar migration as SF220, probably because SF348 has a smaller size with a 3-nt loop 2, and the shortened loop 2 results in a more compact pseudoknot structure even without tertiary interaction with stem 1. Our native PAGE experiment clearly suggests that base triple formation is correlated with the stem 1-loop 2 structural compactness.

### SRV-1 mRNA pseudoknots mechanically unfold cooperatively

**Figure 4:**
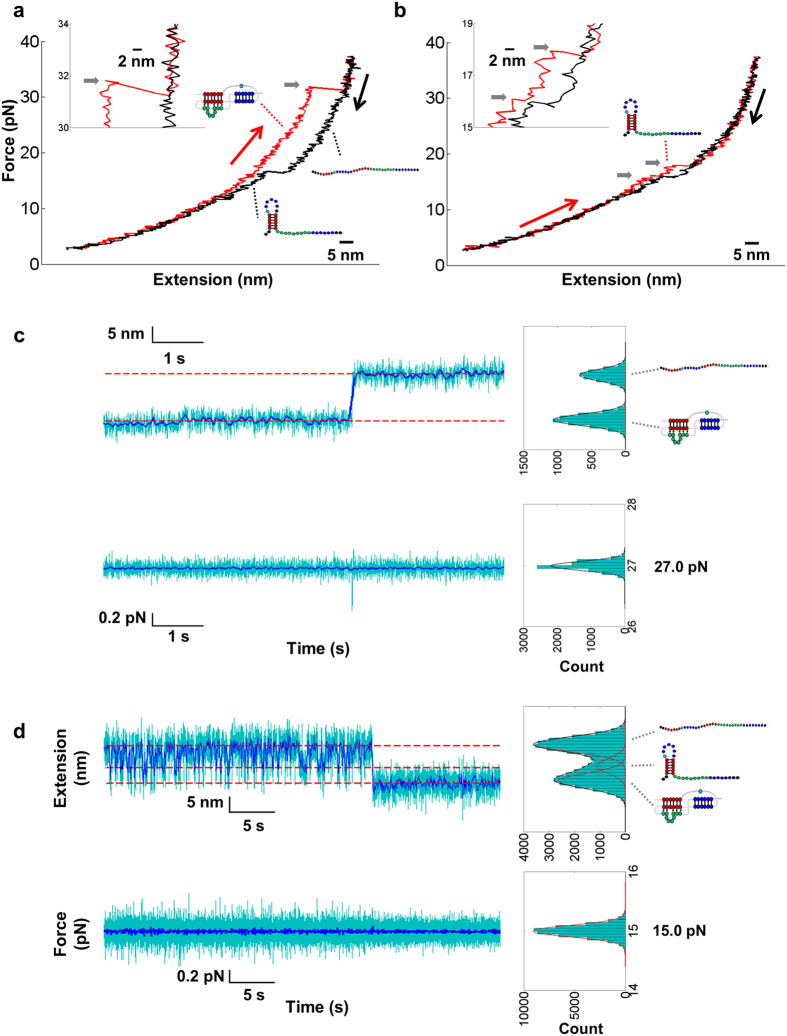
Representative single-mechanical (un)folding traces. (**a**,**b**) Representative force-ramp pulling traces observed for SF209 with a typical one-step unfolding pathway (**a**) and a rarely observed two-step unfolding pathway (**b**). The curves are smoothed to 100 Hz. Red curves are unfolding traces and black curves are refolding traces. The unfolding force is indicated by a horizontal grey arrow. (**c**,**d**) Representative force-jump traces for the one-step unfolding and two-step refolding of SF206 at 27.0 and 15.0 pN, respectively. The 1000 Hz data (cyan) were subsampled to 20 Hz (blue). The dashed lines indicate the Gaussian distribution fitting results. Extensions were binned to 1 nm, and forces were binned to 0.05 pN.

**Figure 5:**
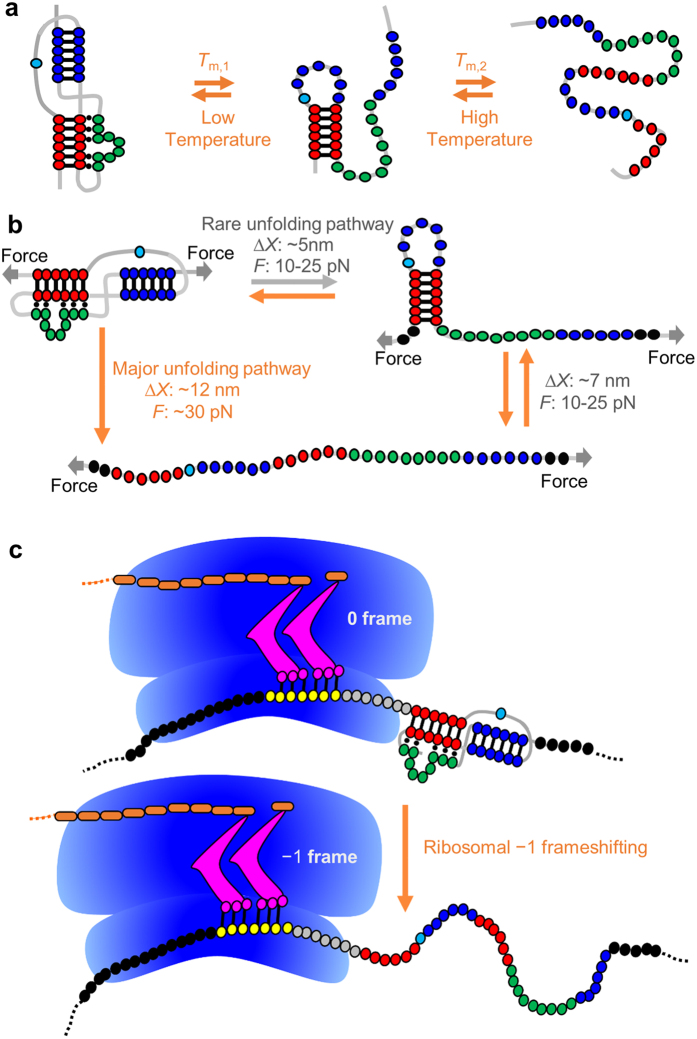
Comparison between thermal and mechanical (un)folding pathways and scheme of ribosomal −1 frameshifting. (**a**) Proposed thermal unfolding and folding pathways (see Fig. 2). (**b**) Proposed single-molecule mechanical unfolding and folding pathways. The stabilizing effect of coaxial stacking between stem 1 (red) and stem 2 (blue), and the base triples formed between stem 1 and loop 2 (green) on the component stems (stem 1 and stem 2) is revealed by analyzing the one-step mechanical unfolding forces. (**c**) Scheme of ribosomal frameshifting from 0 frame to −1 frame. The mRNA slippery sequence and single-stranded spacer within the ribosome are shown in yellow and gray, respectively. Stem 1-loop 2 base triple formation in the mRNA pseudoknot may enhance the local stability of stem 1 and result in enhanced frameshifting. The tRNAs and nascent peptide are shown in magenta and brown, respecitvely. The drawing is not to scale.

To gain insight into the possible determinants of SRV-1 pseudoknot structure for stimulating frameshifting, we carried out single-molecule mechanical unfolding studies using optical tweezers. An H-type pseudoknot structure typically unfolds cooperatively at a relatively high force compared to a hairpin^37^,^40^,^48^,^49^. In our force-ramp experiments, all sequences refolded into pseudoknot structures as indicated by the subsequent unfolding traces with a relatively high unfolding force (Fig. 4a and Supplementary Fig. S4). More than 95% of pulling cycles showed a one-step unfolding transition during stretching with a force loading rate of about 11 pN/s (Fig. 4a and Supplementary Fig. S4 and Table S1). Consistent with the force-ramp experiment, the extension versus time traces in the constant-force experiment^50^ revealed one-step unfolding from pseudoknot to single-stranded conformation (Fig. 4c and Supplementary Figs S6 and S7b). Clearly, thermal and mechanical unfolding show different pathways (Fig. 5a,b). The extension changes of one-step unfolding transition are consistent with the prediction of the extensible wormlike chain (EWLC) model^51^ for unfolding of pseudoknots into stretched single strands (Supplementary Table S1 and Fig. S5). In rare cases, we observed two-step unfolding traces for SF209 (2.0%), SF217 (0.3%), SF220 (1.2%), SF229 (2.8%), and SF348 (5.0%) (see Figs 4b and 5b, and Supplementary Fig. S4j and Table S1). A typical one-step pulling cycle has a large hysteresis, while a typical two-step pulling cycle shows decreased unfolding force and has a decreased hysteresis or is reversible. Varying the force loading rate (5 and 20 pN/s) did not significantly affect the one-step unfolding pathway (see Supplementary Fig. S4 and S5 for example) and kinetics (data not shown).

### Steered Molecular Dynamics (SMD) simulation reveals mechanical unfolding pathway and energetic coupling

**Figure 6:**
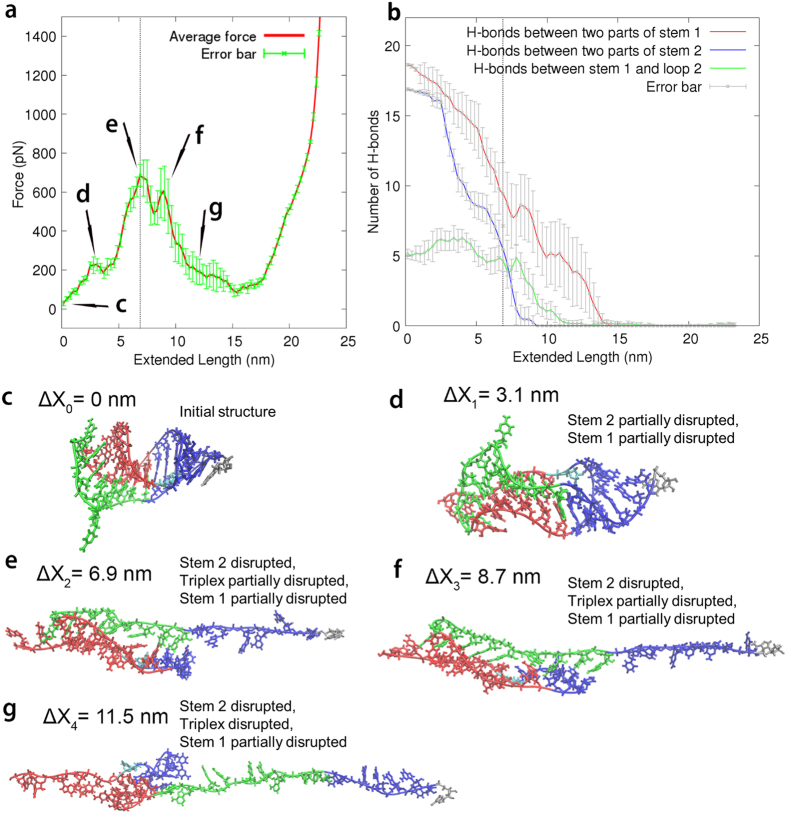
SMD simulation results for SF206. (**a**) An averaged curve of a total of 10 independent pulling simulations. The green error bars are 95% confidence intervals. The end-to-end distance is defined as the distance between the O3′ atom of the 3′ terminal residue A and the O5′ atom of the 5′ terminal residue G. The vertical dotted lines in panels a and b correspond to the highest peak. (**b**) Averaged number of hydrogen bonds formed as a function of extension based on the 10 independent simulations. (**c–g**) Structural snapshots corresponding to the positions in panel a. The snapshots are taken from a representative simulation.

A total of 10 SMD simulations of constant-speed pulling were performed for the “wild-type” SRV-1 pseudoknot (SF206) (Fig. 6). The average pulling force versus extension profile (Fig. 6a) revealed multiple peaks (peaks d, e, f, and g), with the representative structural snapshots shown in Fig. 6d–g. At least one of the two major peaks e and f were present in all 10 simulations, which correspond to the disruption of stem 2 and partial disruption of stem 1-loop 2 triplex and stem 1 (Fig. 6b,e,f). The first small peak (peak d, Fig. 6a) in the force-extension profile is due to the initial disruption of the base pairs in stem 1 and stem 2 (at the two terminal ends of the pseudoknot, Fig. 6b,d). The last small peak g (Fig. 6a) corresponds to the final unfolding stage of the remaining base pairs in stem 1 (Fig. 6b,g). The simulations suggest a common unfolding mechanism, in which stem 2 unfolds first, followed by the unfolding of the triplex and stem 1.

Stem 1 is partially open in early stages, probably because the spring constant in the simulation is larger than that in the pulling experiment (1661 pN/nm in X/Y/Z directions versus 0.06 pN/nm in the pulling direction). A relatively large spring constant in the simulations may cause the local mechanical melting of stem 1 and stem 2 at the terminal ends, which is indeed observed in our simulations but not in the pulling experiment. In addition, due to the computational expense, we used a pulling rate in the simulation (1 nm/ns or 1 m/s) which is much faster than the experimental pulling rate (100 nm/s). Furthermore, the experimental time and spatial resolutions (1–10 ms and ~1 nm) are relatively low compared to those of the smoothed simulation traces (0.3 ns and 0.3 nm). A similar difference between experimental and simulated force-extension profiles was reported previously^39^. Nevertheless, the SMD simulation results revealed the relative mechanical stabilities of the three major parts of the RNA pseudoknot SF206 and provide insight into the microscopic unfolding mechanism.

### SRV-1 mRNA pseudoknots mechanically fold in two steps

More than 99% of the experimental force-ramp refolding trajectories contain two transitions (see Fig. 4a,b and Supplementary Fig. S4 for example). Consistent with the force-ramp experiment, the extension versus time traces in the constant-force experiment^50^ reveal two-step folding traces (Fig. 4d and Supplementary Fig. S6). The first step corresponds to fast and reversible transition between single-stranded and intermediate states with about 6–7 nm extension change (Fig. 4d and Supplementary Fig. S6). The extension change of the single-stranded state to intermediate state transition indicates the formation of a 19-nt hairpin containing stem 1 of the pseudoknots (Figs 4d and 5b and Supplementary Fig. S6).

The second step corresponds to the transition from intermediate state to pseudoknot with 4–5 nm extension change for all the pseudoknots except SF348. For SF348 with a shortened loop 2 (Fig. 1a and Supplementary Fig. S1), we didn’t observe extension change for the transition from intermediate state to pseudoknot (Supplementary Fig. S7a,c). After the formation of the pseudoknot structure at low forces, the extension versus time (constant-force) trajectory is stable without hopping (Fig. 4d), which is consistent with the fact that pseudoknots have relatively high unfolding forces as previously reported^37^,^40^,^48^,^49^. We observed, based on the extension versus time curves (see Supplementary Fig. S6a), that one of the relatively unstable pseudoknots, SF217, has a lifetime of about tens of seconds for the pseudoknot structure at 15–17 pN. The rapid exchange between the single strand and the hairpin conformations suggests that the stem 1 hairpin structure unfolds and folds multiple times before the native pseudoknot structure can form (Fig. 5b). SF348 has a similar folding pattern with a relatively smaller extension change for the transition from the intermediate state to the pseudoknot state (Supplementary Fig. S7a,c) due to the presence of a significantly shortened loop 2 (Fig. 1a and Supplementary Fig. S1). We did not observe the intermediate with stem 2-containing hairpin forming. The formation of stem 2-containing hairpin from single strand would result in an extension decrease of about 8–10 nm at the force range of 10–30 pN, instead of our observed extension decrease of 6–7 nm for the first folding step (Fig. 4d and Supplementary Figs S6 and S7). Taken together, stem 1 forms before stem 2 during mechanical folding (Fig. 5b).

### Minor-groove base triples enhance the mRNA pseudoknot mechanical stability and thereby stimulate frameshifting

**Figure 7:**
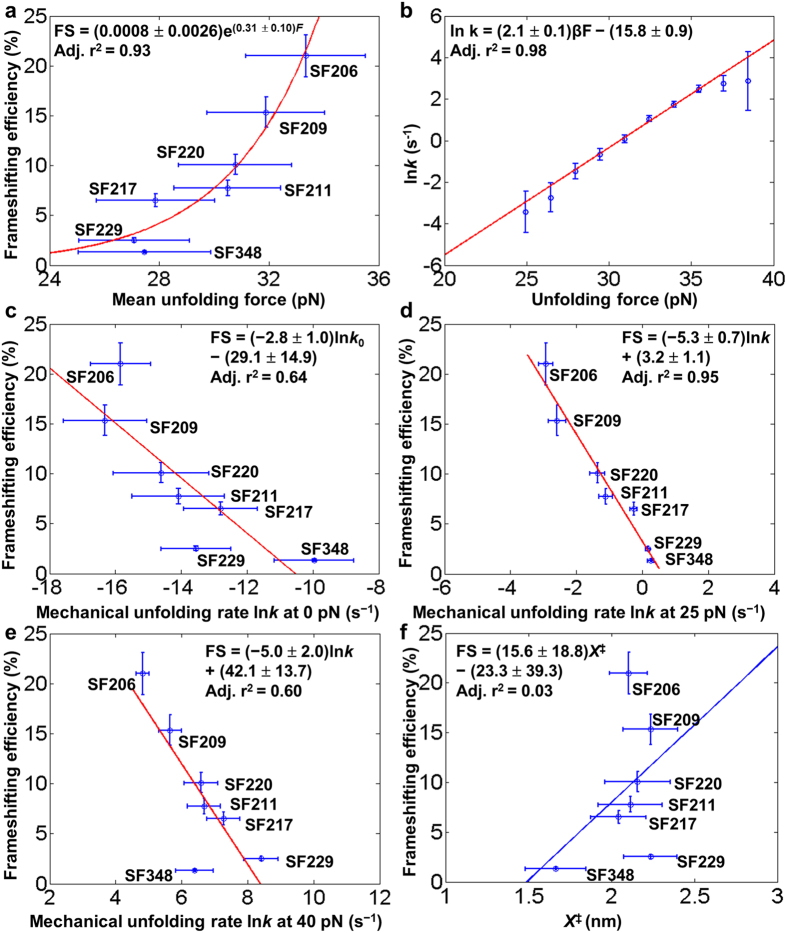
Relationship between mechanical unfolding and frameshifting. (**a**) *In vitro* frameshifting efficiency^19^ versus mean unfolding force. (**b**) Unfolding kinetics of SF206 extracted from force distributions using Dudko’s method^52^. Bell’s model is used to fit the force-dependent unfolding rates to extract rate constants at different forces (*k*) and unfolding transition state positions (*X*^≠^). (**c–e**) *In vitro* frameshifting efficiency^19^ versus unfolding kinetics at the stretching forces of 0, 25, and 40 pN, respectively. The correlation is best at a stretching force of around 25 pN (see Supplementary Fig. S10). (**f**) *In vitro* frameshifting efficiency^19^ has no correlation with mechanical unfolding transition state position *X*^≠^. Error bars of both force and frameshifting efficiency represent standard deviations. Errors of ln*k* and *X*^≠^ are reported as standard errors.

We extracted the pseudoknot one-step unfolding kinetics from unfolding force distributions using Dudko’s method (Supplementary Figs S8 and S9)^52^. Bell’s model (see Supplementary methods) was used to fit the force-dependent unfolding rates to extract the rate constant at zero force (*k*_0_) (Fig. 7b,c). A26C mutation in SF217 is expected to result in the disruption of the A26∙G4-C16 base triple and base stacking among A25, A26 and U27 (Fig. 1c)^18^. We observed, compared to SF206, a relatively large increase (from −15.8 ± 0.9 to −12.8 ± 1.1 s^−1^) in one-step mechanical unfolding rate at zero force, ln*k*_0_ (or ln*k*_(0pN)_) and reduction in mean unfolding force (from 33.3 ± 2.2 pN to 27.9 ± 2.2 pN) (Fig. 7a,c), consistent with the previously observed^19^ decrease in frameshifting efficiency (from 21.0% to 6.6%).

U27C mutation in SF220 is not expected to disrupt the single hydrogen bond formed between U27 and G15 in the U27∙G15-C5 base triple (Fig. 1a,c)^18^. The increase in ln*k*_0_ values (from −15.8 ± 0.9 to −14.6 ± 1.5 s^−1^) and decrease in mean one-step unfolding force (from 33.3 ± 2.2 pN to 30.8 ± 2.1 pN) (Fig. 7a,c), and previously observed^19^ decrease in frameshifting efficiency (from 21.0% to 10.1%) might be due to the unfavorable stacking of C27 with A26 (both with an exocyclic amine group, Fig. 1c). Thus, single-molecule mechanical unfolding studies can reveal the effect of subtle U to C substitution in a loop in a pseudoknot, as has been observed in RNA hairpins containing internal loops in the stems^53^. The relative mechanical stabilities (SF206 > SF220 > SF217) are consistent with the relative gel mobilities observed in the PAGE experiment (Fig. 3), suggesting that gel mobility correlates with structural compactness and mechanical stability for the pseudoknots of the similar size and with the same stem 1 and stem 2.

In SF229, the 9-nt adenine-rich loop 2 is substituted with a pyrimidine-rich sequence (Fig. 1a and Supplementary Fig. S1). Remarkably, compared to SF217 (A26C), SF229 showed only a small decrease in mean unfolding force (from 27.9 ± 2.2 to 27.1 ± 2.0 pN) (Fig. 7a) and a previously observed moderate frameshifting efficiency decrease (from 6.6% to 2.5%)^19^. Our single-molecule data suggest the critical role of A26 in stabilizing the stem 1-loop 2 triplex and the pseudoknot, consistent with our PAGE result (Fig. 3). A mutant with a decreased unfolding force typically corresponds to an increased unfolding rate. However, compared to SF217 (A26C), SF229 showed a decrease in ln*k*_0_ value (from −12.8 ± 1.1 to −13.6 ± 1.1 s^−1^) (Fig. 7c). The opposite trends of mean unfolding force and unfolding rate observed for SF217 and SF229 might imply that, due to the force generation by the ribosome on the mRNA structure^54^,^55^,^56^,^57^, the frameshifting efficiency may correlate better with unfolding kinetics at nonzero forces. Indeed, we observed an improved correlation between frameshifting efficiency and mechanical unfolding rates at forces of 20–30 pN (Fig. 7c–e, Supplementary Fig. S10). It is also possible that a ribosome exerts its helicase activity by directly acting on some of the loop 2 residues. Thus, compared to other SRV-1 pseudoknots with a purine-rich loop 2, SF229 (with a pyrimidine-rich loop 2) may have varied interactions with the ribosome, which may in turn affect the frameshifting efficiency.

SF348 has a significantly shortened loop 2 with only three A residues. Consistent with our thermal melting study (Fig. 2e), SF348 forms a pseudoknot in 200 mM NaCl as revealed by our optical tweezers study (Supplementary Figs S4i,j, S5g, and S7). Similar to SF229 and SF217, SF348 has relatively fast unfolding kinetics with a ln*k*_0_ value of −10.0 ± 1.2 s^−1^ and low mean unfolding force (27.5 ± 2.1 pN) (Fig. 7a,c), and previously observed low frameshifting efficiency (1.3%)^19^, indicating that a shortened loop 2 causes the disruption of the minor-groove base triples. A 3-nt loop 2 may also create steric strain resulting in partial deformation of stem 2 and/or unstacking with stem 1.

Taken together, our results suggest that loop 2 sequence and length can significantly affect pseudoknot mechanical unfolding and frameshifting efficiency through the stabilization of the component stems by energetic coupling. Such a coupling effect of stem-loop interactions in stabilizing stem 1 has been observed in the recent nanopore pseudoknot unzipping experiments^45^.

### The terminal base pair of stem 1 affects the mechanical stability and frameshifting

As revealed by the Cryo-electron microscopy (cryoEM) studies of infectious bronchitis virus (IBV)-rabbit ribosome complexes, the downstream pseudoknot structure is in direct contact with the ribosome at the slippery site^58^. We tested the effect of the terminal Watson−Crick base pair of stem 1 of SRV-1 pseudoknot (Fig. 1a and Supplementary Fig. S1), which is directly in contact with the ribosome (Fig. 5c), on the mechanical unfolding and frameshifting^19^. Consistent with our thermal denaturation data (Fig. 2e) and thermodynamic nearest-neighbor model prediction^42^,^43^,^44^, the mean one-step unfolding forces of SF206, SF209 and SF211 showed the same trend although they are within a small range (33.3 ± 2.2, 31.9 ± 2.1, and 30.5 ± 1.9 pN, respectively) (Fig. 7a and Supplementary Fig. S8). The reported frameshifting efficiencies are 21.0%, 15.3% and 7.8%, respectively, for SF206, SF209, and SF211^19^. The one-step unfolding rates at zero force, ln*k*_0_, of SF206, SF209 and SF211 are −15.8 ± 0.9, −16.3 ± 1.3 and −14.1 ± 1.4 s^−1^, respectively (Fig. 7c). We observed a better correlation between unfolding rate and frameshifting efficiency at nonzero forces for SF206, SF209, and SF211 (Fig. 7c–e and Supplementary Fig. S10). As applying stretching force destabilizes the RNA structures, our results imply that stem 1 is destabilized by the translocating ribosome which has helicase activity^57^ in unwinding mRNA structures. Thus, the 5′ terminal base pairs in stem 1, which is in direct contact with the translocating ribosome, can modulate mechanical stability of the pseudoknot and thus frameshifting efficiency, consistent with previous studies on the frameshift-inducing HIV-1 hairpin^17^ and antisense oligonucleotides^23^. Thus, frameshifting can be stimulated by enhancing the local stability of the base pairs positioned at the mRNA entrance channel of the ribosome (Fig. 5c) through (i) directly improving the local base pairing interactions and (**ii**) indirect energetic coupling by triplex formation.

### One-step unfolding rupture force and unfolding rate correlate with *in vitro* frameshifting efficiency

We compared the mean one-step unfolding forces (Fig. 7a and Supplementary Fig. S8) to the *in vitro* frameshifting efficiency values measured previously^19^. The mean one-step unfolding forces have a positive correlation with *in vitro* frameshifting efficiencies. The unfolding rate constant extracted from Bell’s model showed an inverse correlation with *in vitro* frameshifting efficiency (Fig. 7a–e, Supplementary Figs S9, and S10 and Tables S1 and S2). Importantly, we note that the inverse correlation between the one-step unfolding rate and frameshifting efficiency was clearly observed in a broad range of forces from 0 to 40 pN (Fig. 7c–e and Supplementary Fig. S10). An improved correlation was observed when the forces were closer to the unfolding forces (15–35 pN), consistent with the fact that a ribosome is a force-generating molecular motor with helicase activity^54^,^55^,^56^,^57^. For example, by applying mechanical stretching force on the translating *E. coli* ribosome and the 3′ end of an unstructured mRNA, it has been estimated that an actively translating *E. coli* ribosome can resist a force of about 13 pN^55^. It is in reasonable agreement with an improved correlation between frameshifting efficiency and SRV-1 mRNA pseudoknot unfolding kinetics when the forces are closer to the unfolding forces (15–35 pN). The discrepancy of the force magnitudes (resistant force of 13 pN for *E. coli* ribosome versus SRV-1 mRNA pseudoknot unfolding force of 15–35 pN) may be due to the reasons shown below. (**i**) As a ribosome is a large RNA-protein complex, it is likely that the resistant force of the ribosome is highly dependent on the points of the applied force. (**ii**) The frameshifting efficiencies for SRV-1 pseudoknots were previously measured for an eukaryotic ribosome (using a commercially available *in vitro* translation system)^19^. (**iii**) During ribosomal frameshifting, a ribosome in contact with a downstream frameshifting stimulatory mRNA structure can pause seconds to minutes and change its conformation^30^,^31^,^32^,^33^,^34^ to facilitate the unwinding of highly stable mRNA structures including pseudoknots. The distances between the folded state and unfolding transition state (*X*^‡^) showed no correlation with frameshifting efficiencies and are about 2 nm for all pseudoknots (Fig. 7f), which is consistent with the previous results on the unfolding of human telomerase RNA and other pseudoknots^38^,^48^.

Several factors may cause the relatively poor correlation between the one-step unfolding rate and frameshifting efficiency at stretching forces outside the range of 20–30 pN. (**i**) The directions and points of the applied force are probably not the same as those of the force generated by a ribosome (Fig. 5b,c). (**ii**) The triplex and pseudoknot structure can form in the absence of magnesium ions^18^, and in our single-molecule pulling experiments, no magnesium ion is present. However, magnesium ions may affect the (un)folding dynamics of the pseudoknots differently. (**iii**) The single-stranded spacer is slightly different for the pseudoknots with the mutations in stem 1 and loop 2, which may affect the ribosome-mRNA interactions, although the single-stranded spacers may not affect the downstream pseudoknot (un)folding significantly. (**iv**) Bell’s model for the extraction of unfolding kinetics from unfolding force distribution may not be accurate with the forces far away from the unfolding force range (20–40 pN, see Supplementary Fig. S8).

## Conclusions

Our equilibrium thermal melting data showed no obvious correlation between thermal stability and *in vitro* frameshifting efficiency measured previously^19^. The absence of a correlation is consistent with previous observations^37^ and is likely due to the fact that the formation of stem 2 and minor-groove triples (formed between stem 1 and loop 2) in a pseudoknot may exert a coupling effect in stabilizing stem 1 as has been observed in the recent nanopore pseudoknot unzipping experiments^45^. The thermodynamic stabilization effect due to the coaxial stacking between stem 1 and stem 2 and stem 1-loop 2 bases triple formation are not observed in the thermal denaturation, because under the global temperature perturbation, the base triples and stem 2 melt before stem 1 (Fig. 5a).

In contrast to a stepwise thermal unfolding process, the applied mechanical stretching force facilitates cooperative mechanical unfolding of the SRV-1 pseudoknots (Fig. 5b). Although, the directions and points of the applied force may not be the same as those of the force generated by a ribosome (Fig. 5b,c), our single-molecule mechanical unfolding results provide unique insights into how compaction of a pseudoknot through coaxial stacking and stem-loop interactions may stabilize the component stems. The single-molecule mechanical unfolding results of SRV-1 pseudoknots suggest that the cooperative mean unfolding force has a positive correlation with −1 frameshifting efficiency when the slippery sequence and the spacer are maintained largely constant, consistent with previous studies^35^,^37^. However, the possible correlation between mechanical unfolding rate and frameshifting efficiency has not been extensively investigated, due to a limited number of mutants studied or a small number of pulling traces obtained for the native pseudoknots^35^,^36^,^37^,^39^. We showed in this study that the cooperative mechanical unfolding rates of the seven SRV-1 pseudoknots studied are inversely proportional to frameshifting efficiencies in a wide range of stretching forces. An improved correlation was observed at the stretching forces of 15–35 pN, consistent with the fact that ribosome is a force-generating molecular motor with helicase activity^54^,^55^,^56^,^57^.

Our studies suggest that the frameshifting mRNA pseudoknot structures stabilized by highly cooperative secondary and tertiary interactions may serve as kinetic barriers against the forward translocation of ribosome and reduce the speed of translation, and thus increase the probability of −1 frameshifting. The minor-groove stem 1-loop 2 base triples in mRNA pseudoknots may be generally employed for stimulating translational recoding in other viruses and cells^8^,^9^,^15^,^59^. Our studies provide detailed insights into the determinants of a pseudoknot structure affecting frameshifting efficiency, which may help us understand and manipulate the −1 frameshifting process by designing pseudoknot-binding ligands^12^,^13^,^60^.

## Materials and Methods

### *In vitro* frameshifting assay

The *in vitro* frameshifting efficiency values were taken from a previous study^19^ that made use of a commercially available *in vitro* translation system (rabbit reticulocyte lysate (Promega)) and wherein the *in vitro* translated protein products were resolved and quantified by SDS-PAGE.

### Thermal denaturation experiments

The HPLC-purified RNA oligonucleotides for UV-absorbance-detected thermal denaturation experiments were purchased from Sigma. The ensemble thermal denaturation experiments for the pseudoknots and a control hairpin were conducted using a Shimadzu 2550 spectrometer. The control hairpin (residues 1 to 19) is a truncated form of the pseudoknot SF206 (residues 1 to 34) (see Fig. 1a and Supplementary Fig. S1). The RNA oligonucleotide sample concentrations were 3 μM. All pseudoknots and the control hairpin were heated at 85 °C then slowly cooled to 15 °C in more than 2 hours before the thermal melting experiments. The temperature ramping rate was 0.5 °C/min. Absorption values at 260 nm were recorded. The buffers contained 20 mM HEPES, 0.1 mM EDTA, pH 7.3 with varying NaCl concentrations (0 mM, 200 mM or 1 M NaCl). The measurement for each sample at each buffer condition was repeated three times. The melting temperatures were obtained from dA/dT versus temperature curves and presented as mean ± standard deviation.

### Native PAGE

RNA oligonucleotides (with residues 1–34 for the pseudoknots or residues 1–19 for the control hairpin, see Fig. 1a) for native PAGE were annealed in the buffer with 200 mM NaCl, 20 mM HEPES, 0.1 mM EDTA, pH 7.3. The pseudoknot solutions were heated at 95 °C for 5 min, then slowly cooled to room temperature for 3 h and equilibrated at 4 °C overnight. The control hairpin solution was heated at 95 °C for 5 min, then snap-cooled on ice and equilibrated at 4 °C overnight. Each sample was mixed with 6× loading dye (QIAGEN) and diluted with buffer to have a final 1× loading dye and a final RNA concentration of 1 μM. Then 20 μL of each sample was loaded onto a 15% native polyacrylamide gel with 1× TBE (89 mM Tris/Borate, 2 mM EDTA, pH 8.3) running buffer. The gel was run at 250 V for 10 h at 4 °C, and was post-stained with ethidium bromide.

### Sample preparation for single-molecule optical tweezers experiments

Single-molecule constructs (see Fig. 1a,b) were made as described previously^37^,^48^, using the recombinant pSFCASS5 plasmids containing the pseudoknot sequences^19^. The ~1160-nt RNA molecules were made by PCR amplification of the plasmids followed by *in vitro* transcription by T3 RNA polymerase (Promega). The RNAs contain a 475-nt upstream sequence, a 2-nt single-stranded linker (GA), the pseudoknot sequences, a downstream 2-nt single-stranded linker (AU), and a 630-nt downstream sequence. The RNAs were annealed with complementary strands of PCR-generated 475-bp and 630-bp double-stranded DNAs (dsDNAs) to generate RNA/DNA hybrid handles (handle A and handle B). T4 DNA polymerase (NEB) was used to label the 3′ end of the DNA strand of handle A by introducing biotin-16-dUTP (Roche). Handle B was labeled at the 5′ end of the DNA strand by digoxigenin labeled primer during PCR. 1.8-μm Streptavidin coated polystyrene beads were purchased from Spherotech. Recombinant protein G (Thermo Scientific) was coated on 3-μm carboxyl coated polystyrene beads (Spherotech) using EDC (Sigma) and sulfo-NHS (Thermo Scientific). Dimethyl pimelimidate (Thermo Scientific) was used to cross-link anti-digoxigenin antibody to the protein G-coated beads. Only appropriately annealed DNA/RNA hybrids are able to form a tether with both types of beads.

### Optical tweezers experiments

Single-molecule measurements were performed using a dual-beam Minitweezers^61^. The anti-digoxigenin-coated polystyrene bead was trapped in the optical trap and the streptavidin-coated polystyrene bead was held on a micropipette by suction (Fig. 1b). In a force-ramp experiment, the force was changed by moving the optical trap at a force loading rates of about 11 pN/s, with a constant stiffness of about 0.06 pN/nm. The force was maintained by a 200 Hz electronic force feedback in constant-force experiments. The data acquisition rate was 1000 Hz. All of the optical tweezers experiments were carried out at 22 ± 1 °C. The buffer contains 200 mM NaCl, 10 mM Tris-HCl, 0.1 mM EDTA, pH 7.3. For each sample, data were collected for at least 3 individual tethers.

## Additional Information

**How to cite this article**: Zhong, Z. *et al*. Mechanical unfolding kinetics of the SRV-1 gag-pro mRNA pseudoknot: possible implications for −1 ribosomal frameshifting stimulation. *Sci. Rep.* **6**, 39549; doi: 10.1038/srep39549 (2016).

**Publisher's note:** Springer Nature remains neutral with regard to jurisdictional claims in published maps and institutional affiliations.

## Supplementary Material

Supporting Information
