# Supplementary material for: Mechanical unfolding kinetics of the SRV-1 gag-pro mRNA pseudoknot: possible implications for −1 ribosomal frameshifting stimulation
Source: Sci Rep. 2016 Dec 21;6:39549. doi: 10.1038/srep39549 (PMC5175198; doi:10.1038/srep39549)
Supplement: Supporting Information [file srep39549-s1.pdf]

Supporting Information

Mechanical unfolding kinetics of the SRV-1 gag-pro mRNA pseudoknot: possible implications for −1 ribosomal frameshifting stimulation

Zhensheng Zhong,[1] Lixia Yang,[1] Haiping Zhang,[2] Jiahao Shi,[1] J. Jeya Vandana,[1] Do Thuy Uyen Ha Lam,[1,3] René C. L. Olsthoorn,[4] Lanyuan Lu,[2] and Gang Chen[1]

[1]Division of Chemistry and Biological Chemistry, School of Physical and Mathematical Sciences, Nanyang Technological University, 21 Nanyang Link, Singapore 637371

[2]School of Biological Sciences, Nanyang Technological University, 60 Nanyang Drive, Singapore 637551

[3]St Andrew's Junior College, 5 Sorby Adams Drive, Singapore 357691

[4]Leiden Institute of Chemistry, Leiden University, P.O. Box 9502, 2300 RA Leiden, The Netherlands

Correspondence should be addressed to G.C.: Tel: +65 6592 2549; Fax: +65 6791 1961; Email: RNACHEN@ntu.edu.sg

SUPPLEMENTARY METHODS

Single-molecule data analysis. For force-ramp data, unfolding traces were characterized and unfolding rupture force and extension changes of 1-step unfolding transitions were measured. An approach described by Dudko *et al*. was used to extract the unfolding kinetics from rupture force distributions.[1]

$$k(F) = \frac{\dot{F}(F)\cdot p(F)}{\int_F^\infty p(f)df} = \frac{h_k \dot{F}(F_0+(k-1/2)\Delta F)}{(h_k/2+\sum_{i=k+1}^{N} h_i)\Delta F} \qquad (1)$$

where $\dot{F}(F)$ is the force loading rate at force F, $p(F)$ is the probability density of unfolding events at force F, $\int_F^\infty p(f)df$ is the probability of unfolding events above F, h_k is the height of kth bin in the unfolding force distribution histogram that start at F_0, ΔF is the bin size of the histogram, i and k are the bin numbers, and N is the total bin number of the histogram. Standard errors were estimated as described previously.[2]

Bell's model (Eq. **2**) was used to fit the force-dependent kinetics.[3]

$$\ln k(F) = \ln k_0 + \beta F X^{\ddagger} \qquad (2)$$

Here, $k(F)$ is the force-dependent reaction rate, k_0 (or $k_{(0\ \text{pN})}$) is the reaction rate at zero force, $X^{\ddagger}$ is the extension change from the initial state of the reaction to the transition state, β is equal to $(k_B \cdot T)^{-1}$, in which k_B is the Boltzmann constant, and T is absolute temperature (295 K). More complex models[1] were also applied to fit the force-dependent kinetics data but did not yield reliable fitting parameters.

For constant-force data, the distributions of the extension values were fit with Gaussian model to get the extension change values upon (un)folding.[4] The extensible wormlike chain model (EWLC) (Eq. **3**) was used to convert the extension change values to number of nucleotides unfolded,[5]

$$F = \frac{k_B T}{P}\left[\frac{1}{4\left(1-\frac{\Delta X}{L_0}+\frac{F}{K}\right)^2} - \frac{1}{4} + \frac{\Delta X}{L_0} - \frac{F}{K}\right] \qquad (3)$$

Here, k_B is Boltzmann constant, T is absolute temperature (295 K), P is persistence length, L_0 is contour length, and K is stretch modulus. For ssRNA, L_0, P and K are 0.59 nm per nucleotide, 1 nm, and 1500 pN, respectively,[6-10] The lengths of the SRV-1 pseudoknot based on the NMR structure of SF206 (PDB 1E95) (4 nm)[11] and the diameter of a hairpin (2 nm) were taken into consideration.

Replica Exchange Molecular Dynamics (REMD) simulation of thermal unfolding process. Thermal unfolding of the SRV-1 RNA pseudoknot SF206 was simulated by REMD.[12,13] Simulations were carried out using the Gromacs program with the AMBER-99SB force field.[14] The starting structure for the simulations was based on the NMR structure of the "wild-type" SRV-1 pseudoknot SF206 (PDB: 1E95) with residues 1-34 plus a 3' terminal residue A (see Fig. 1a).[11] We chose the third structure (out of a total of 15 deposited structures) which has the smallest root mean squared deviation (RMSD) to the average structure. A total of 34 sodium counter ions were added to neutralize the pseudoknot. The system was solvated in a box of TIP3P water. The water box (containing 63946 water molecules) was made sufficiently large (10 nm × 8 nm × 8 nm) to prevent the unfolded RNA encountering its periodic image. Periodic boundary conditions were used and the Particle mesh Ewald method (PME) method[15] was

implemented to calculate the electrostatic interactions. The energy of van der Waals non-bonded interactions was evaluated using a 10 Å cutoff. The LINCS algorithm was applied to the covalent bonds involving hydrogen atoms. The system was subjected to energy minimization to optimize the initial geometry. The standard molecular mechanics steepest decent algorithm was used. A short 100 ps equilibration with position restraints on all atom was conducted after the energy minimization.

The REMD simulations with the temperature ranging from 310 to 511.7 K were performed for the SRV-1 RNA pseudoknot SF206. The systems were equilibrated for 1 ns at the respective temperatures at the isothermal-isochoric condition with the modified Berendsen (V-rescale) thermostat [16]. Finally, the REMD simulations were at the isothermal-isobaric condition with the Parrinello-Rahman barostat[17,18] and the modified Berendsen (V-rescale) thermostat. The total simulation time for REMD is 75 ns with 140 replicas, and the final average exchange rate is about 0.3. The temperature of the replicas is determined by an online REMD temperature generator [19]. Only the last 35 ns of each of the REMD trajectory were used for analysis. The two dimensional free energy plots were calculated from the Boltzmann inversion formula $\Delta G = -RT\text{In}\left(\frac{\rho}{\rho_0}\right)$, using the hydrogen bond (H-bond) numbers of stem 1 and stem 2 as two reaction coordinates. Here, R is the gas constant, and T is 300 K. The density ρ is the conformational distribution density for a grid defined by two H-bond numbers, and ρ_0 is the corresponding normalization term calculated from an idea gas distribution. Free energy values were computed for the grids of the two reaction coordinates, where zero H-bond number means the complete unfolding of the stem and the fully folded structure corresponds to the maximum H-bond number.

Steered Molecular Dynamics (SMD) simulation of the mechanical unfolding process. Mechanical unfolding of the SRV-1 RNA pseudoknot SF206 was simulated by Steered Molecular Dynamics (SMD).[20-22] All of the simulations were carried out using the Gromacs program with the AMBER-99SB force field.[14,23] Sodium ions and chloride ions with a final concentration of 200 mM were added using the Gromacs program tool.[24] The system was solvated in a box of TIP3P water.[25] To accommodate the completely stretched pseudoknot, the water box (containing 70,800 water molecules) is asymmetric with the dimensions of 60 nm × 6 nm × 6 nm. Periodic boundary conditions were used and the PME[15] was implemented to calculate electrostatic interactions. The energy of van der Waals non-bonded interactions were evaluated using a 14 Å cutoff. The LINCS algorithm was applied to the covalent bonds involving hydrogen atoms.[26] The starting structure for the SMD simulation was the same as in the REMD simulations. The system was equilibrated for 1 ns with only the position restraint on the O5' atom of the 5' terminal G residue at 295 K. The equilibrium and SMD simulation were all performed in an isothermal-isobaric ensemble with a fixed temperature of 295 K and a pressure of 1 atm.

A standard SMD protocol was used to stretch the pseudoknot, similar to the works of White *et al*. and Bergues-Pupo *et al*..[22,27] An artificial spring mimicking the optical tweezers pulling experiment was applied to the O3' atom of the 3' terminal residue A. The spring constant was 4152.5 pN/nm. The pulling speed was 1 nm/ns, which resulted in a total simulation time of 24 ns to unfold the pseudoknot. We aligned the pseudoknot to make sure that the straight line between the O3' atom of 3' terminal residue A and the O5' atom of the 5' terminal residue G is parallel to the X axis. The direction of pulling was along the X axis of the simulation box. The O5' atom of the 5' terminal residue G was fixed (by a position restraint with spring constant 1661 pN/nm in

X/Y/Z directions) throughout the SMD simulations. The simulated pulling forces were calculated every 2 fs. A total of ten simulations were performed for SF206. The numbers of H-bonds were calculated every 4 ps. The corresponding curves were smoothed by computing the averages for 80 equally spaced intervals, with the bin width ~0.3 ns (or ~0.3 nm of extension) for each simulation. The means of the intervals were used to plot the simulated profiles using natural cubic splines.

Table S1. Summary of the force-ramp pulling experiments[a]

Molecule	Number of 1-step unfolding traces	Number and occurrence frequency of 2-step unfolding traces	Extension change of 1-step unfolding transitions (nm)	Nucleotides unfolded for 1-step unfolding transitions[b]	Mean 1-step unfolding force (pN)	Reported *in vitro* frameshifting efficiency (%)[c]
SF206	276	0 (0%)	12.3 ± 0.9	32.9 ± 1.8	33.3 ± 2.2	21.0
SF209	298	6 (2.0%)	12.5 ± 1.1	33.5 ± 2.3	31.9 ± 2.1	15.3
SF211	247	0 (0%)	12.5 ± 0.9	33.7 ± 1.9	30.5 ± 1.9	7.8
SF217	368	1 (0.3%)	12.6 ± 1.0	34.4 ± 2.0	27.9 ± 2.2	6.5
SF220	245	3 (1.2%)	13.1 ± 1.0	35.0 ± 1.9	30.8 ± 2.1	10.1
SF229	315	9 (2.8%)	12.4 ± 0.9	34.2 ± 2.0	27.1 ± 2.0	2.5
SF348	192	10 (5.0%)	9.2 ± 0.9	27.4 ± 1.7	27.5 ± 2.4	1.3

[a] All uncertainties are reported as standard deviations.

[b] Calculated using EWLC model. The end-to-end distance (4 nm) of the pseudoknots is considered. All pseudoknots contain 34 nucleotides, except that SF348 has 28 nucleotides.

[c] The frameshifting efficiency values were calculated based on the results reported by Olsthoorn *et al*.[28] The standard deviations are about 10% of the mean values.

Table S2. Summary of one-step mechanical unfolding kinetic parameters at zero force from force-ramp experiment[a]

Molecule	$\ln k_0$ (s^{-1})	$X^{\ddagger}$ (nm)	Adjusted r^2
SF206	−15.8 ± 0.9	2.1 ± 0.1	0.98
SF209	−16.3 ± 1.3	2.2 ± 0.2	0.96
SF211	−14.0 ± 1.4	2.1 ± 0.2	0.96
SF217	−12.8 ± 1.1	2.0 ± 0.2	0.95
SF220	−14.6 ± 1.4	2.2 ± 0.2	0.96
SF229	−13.6 ± 1.1	2.2 ± 0.2	0.97
SF348	−10.0 ± 1.2	1.7 ± 0.2	0.93

[a] All uncertainties are reported as standard errors.

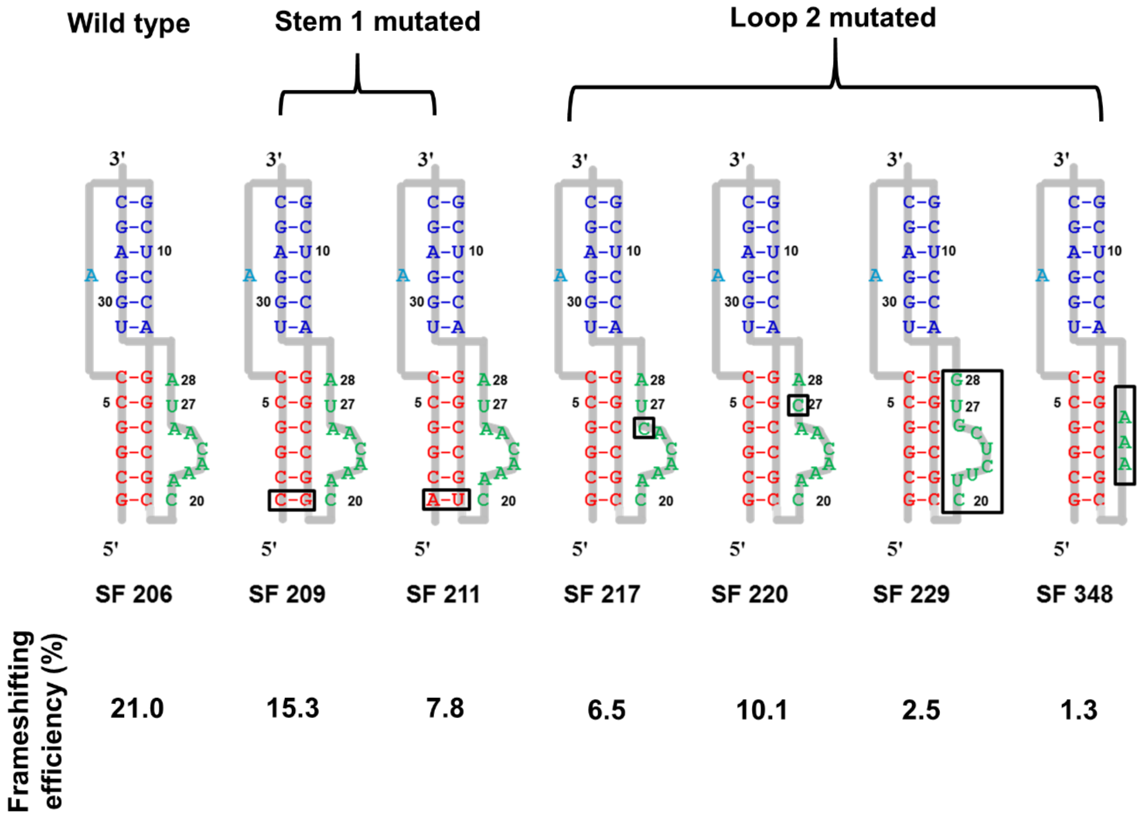

Figure S1. The pseudoknot structures studied in this paper. The *in vitro* frameshifting efficiency values were measured previously with the standard deviations about 10% of the mean values.[28]

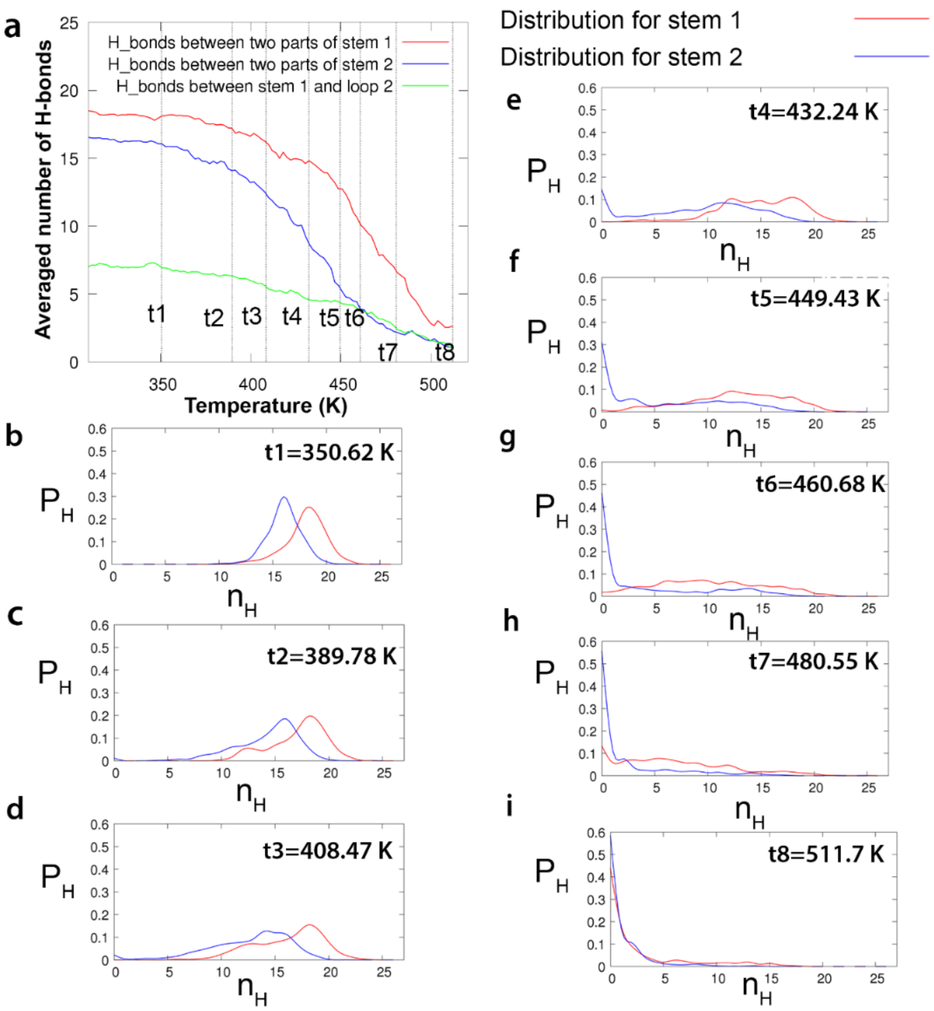

Figure S2. Replica exchange molecular dynamics (REMD) simulations for SF206. (**a**) Plot of the average number of H-bonds (n_H) versus temperature in the corresponding replicas of different temperatures. Only the H-bonds between two bases are counted. The distance and angle cutoff for the H-bonds are 3.5 Å between two heteroatoms and 150°, respectively. (**b-i**) the plots illustrate the H-bond number distribution (P_H) of stem 1 (red curve) and stem 2 (blue curve) at temperatures 351, 390, 408, 432, 449, 461, 481, and 511 K, respectively. The distribution is the percentage calculated by n_{bin}/N_{total}, where N_{total} is the total simulation frame number, and n_{bin} is the frame number for a particular bin of H-bond number. Zero number of H-bonds corresponds to a completely unfolded stem. At 351 K (panel **b**), the single distribution peak is close to the native structure with around 16 and 18 hydrogen bonds for stem 2 and stem 1, respectively. At 390 K (panel **c**), the major distribution peak is still around 16 and 18 H-bonds for stem 2 and stem 1, respectively. However, the minor populations of non-native structures of both stems appear at 390 K. Between 408 and 461 K (panels **d-g**), the distribution at zero H-bond of stem 2 gradually becomes the major peak. At temperatures between 481 and 512 K (panels **h-i**), a new distribution peak with zero H-bond in stem 1 emerges and gradually becomes the major peak.

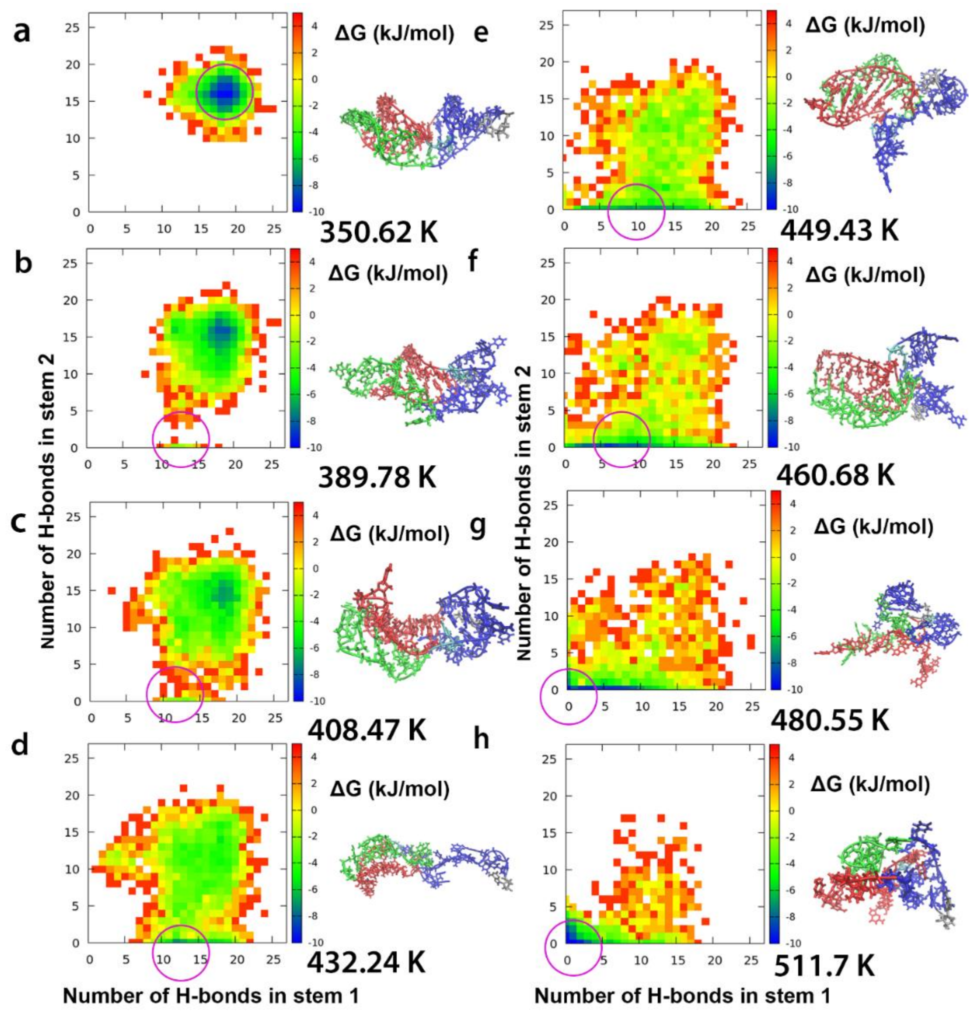

Figure S3. Free energy plots and structural snapshots obtained from the REMD simulations for SF206. The structural snapshots demonstrate that upon increasing the temperature, stem 2 melts before stem 1. The two-dimensional free energy plot is shown on the left side of each panel. The two reaction coordinates are the numbers of base-base H-bonds formed within stem 1 and stem 2, respectively. The color scheme illustrates the free energy values at 300 K obtained from Boltzmann inversion of the simulation population distributions of H-bonds. Shown on the right side of each panel is the structural snapshot from a selected region marked by a pink cycle in the corresponding free energy plot. Panel **a** shows the native state. The structural snapshots shown in panels **b-e** are the intermediate state with stem 2 completed disrupted and stem 1 intact, which becomes an energetically favourable and most populated state at 449 K (see panel **e**). Stem 1 starts to unfold upon further increasing temperature (panel **f**), and the state with both stems unfolded becomes dominant at 481 K (panel **g**). At 512 K, the structure is completely unfolded (panel **h**).

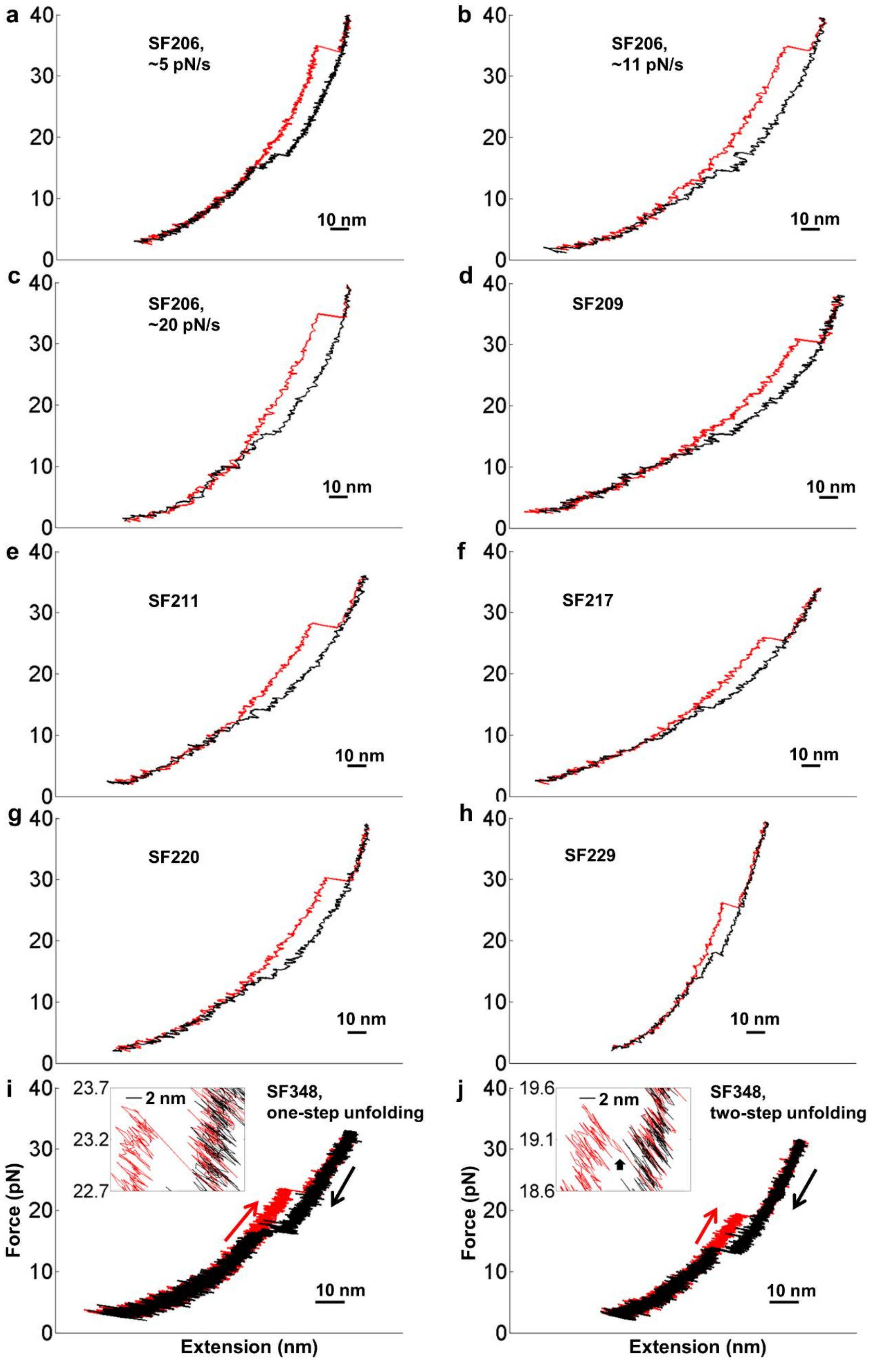
a
SF206,
~5 pN/s
b
SF206,
~11 pN/s
c
SF206,
~20 pN/s
d
SF209
e
SF211
f
SF217
g
SF220
h
SF229
i
SF348,
one-step unfolding
2 nm
23.7
23.2
22.7
j
SF348,
two-step unfolding
2 nm
19.6
19.1
18.6
10 nm
Force (pN)
Extension (nm)
0
10
20
30
40

Figure S4. Representative force-ramp traces. The force (un)loading rate is at about 11 pN/s except for panels a (~ 5 pN/s) and c (~20 pN/s). The data acquisition rate is 100 Hz, except for panels **i** and **j** (1000 Hz). Typically a one-step unfolding transition is observed. A rarely observed two-step unfolding trace is shown in panel **j**. The vertical black arrow in panel **j** indicates the presence of an unfolding intermediate state.

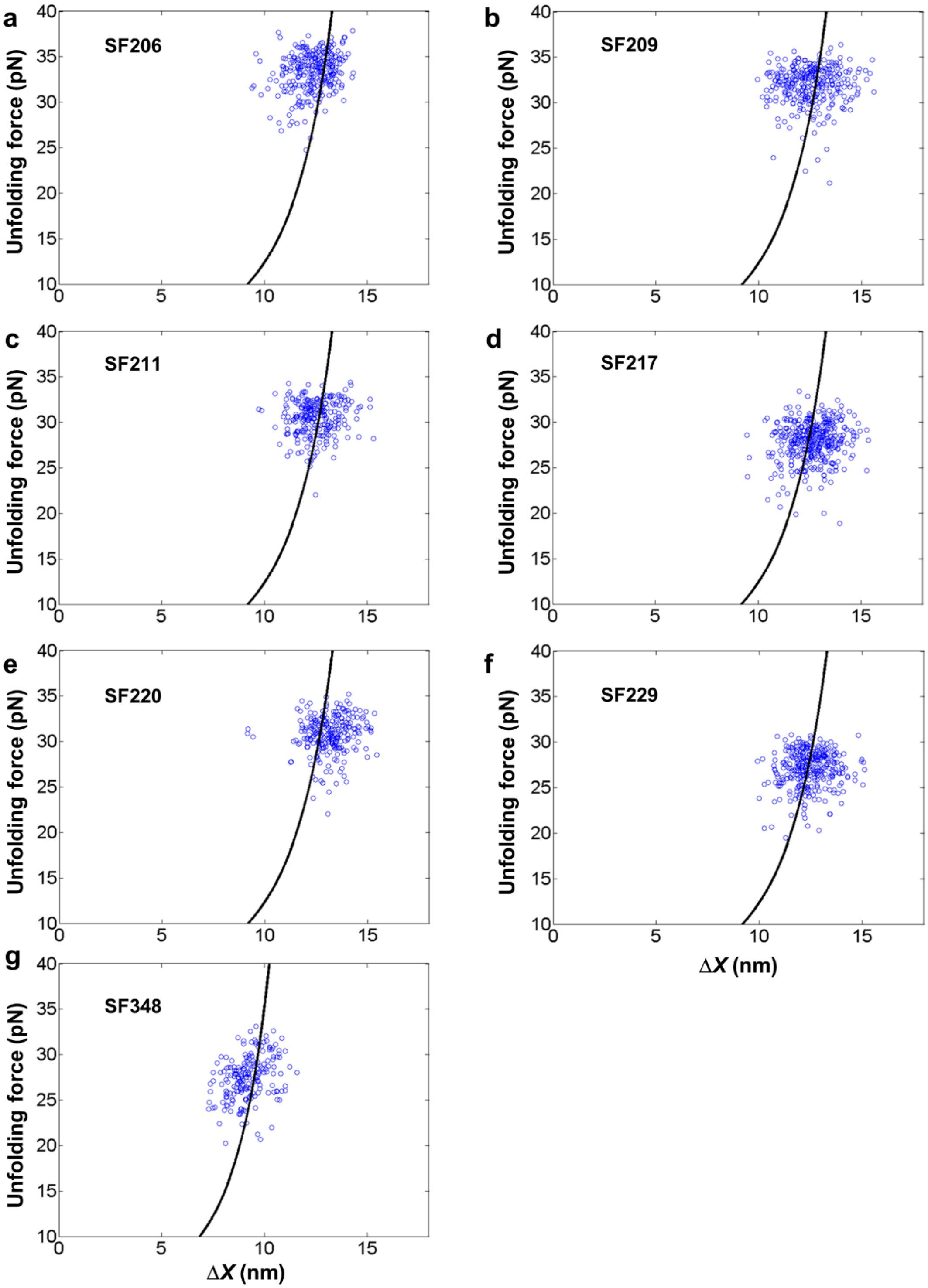

Figure S5. Rupture force versus extension change of one-step unfolding transitions obtained from force-ramp experiment. The predicted EWLC curves are shown for the pseudoknot to single strand transition. The end-to-end distance of the pseudoknot (4 nm) is taken into consideration.

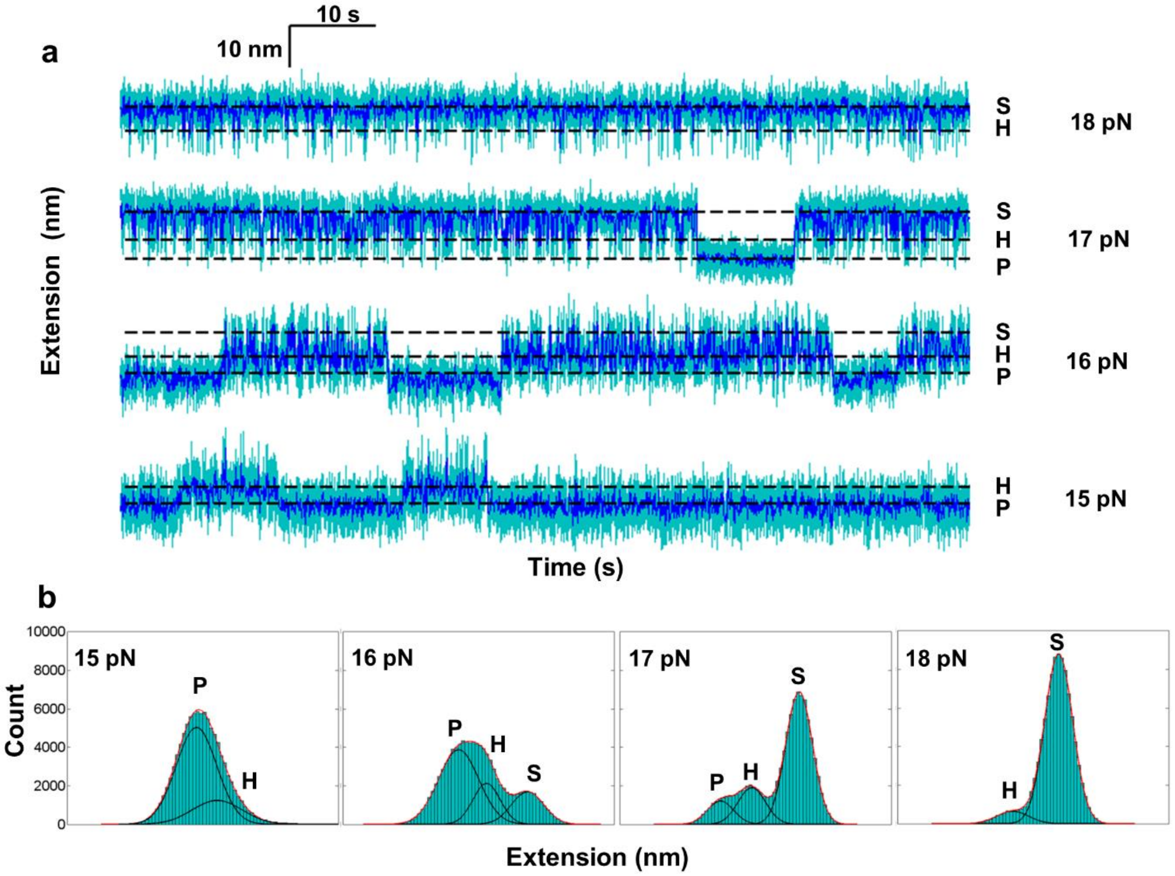

Figure S6. (a) Representative constant-force traces observed for SF217. The 1000 Hz data (cyan) were subsampled to 20 Hz (blue). The dashed lines indicate the Gaussian distribution fitting results in panel **b**. The letters S, H and P represent ssRNA, hairpin and pseudoknot, respectively. At a relatively high force (18 pN), the hairpin structure has a rapid exchange with the single-stranded conformation. The pseudoknot structure is stabilized upon lowering the force to 16-17 pN, and unfolds into the dynamic ensemble of single-stranded and hairpin conformations. At a relatively low force (15 pN), the pseudoknot conformation is further stabilized, and rarely unfolds into the hairpin conformation. Consistently, in the force-ramp experiment, the two-step and one-step unfolding forces for SF217 are in the range of 15-20 pN and > 20 pN, respectively. Thus, the constant-force and force-ramp data of S217 suggest that the energy barrier of pseudoknot to hairpin transitions is higher and stiffer than that of hairpin to pseudoknot.[29] **(b)** Histograms of extensions in panel **a**. Extensions were binned to 1 nm. The histograms were fitted using Gaussian functions.

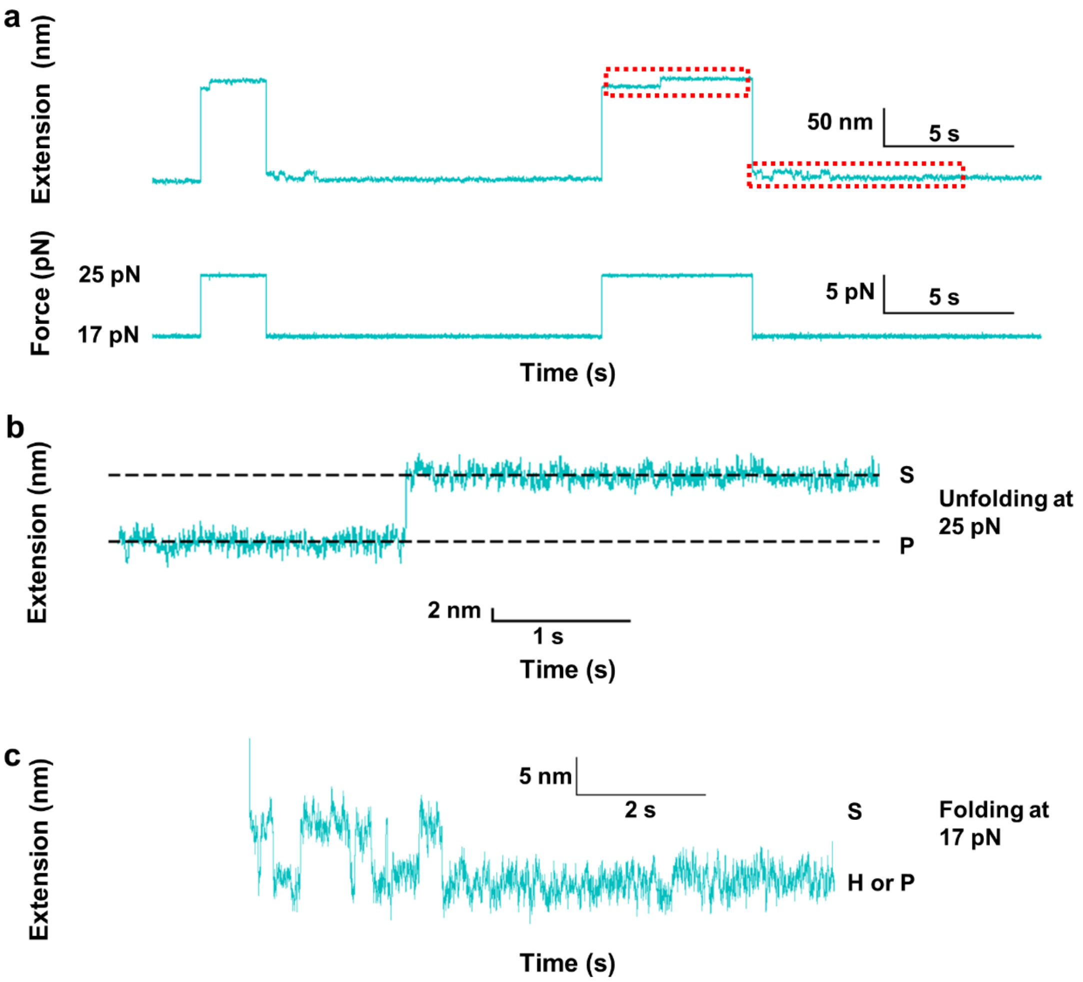

Figure S7. Representative constant-force traces observed for SF348. The 1000 Hz data (cyan) were subsampled to 20 Hz (blue). The letters S, H and P represent ssRNA, hairpin and pseudoknot, respectively. **(a)** Representative force-jump cycles for pseudoknot SF348 with a zoom-in view for the boxed regions shown in panels **b** and **c**. (**b**) A one-step unfolding transition at 25 pN. **(c)** As the extension change between intermediate state and pseudoknot sate is very small for SF348, we did not observe the transition directly. However, the refolding trace of SF348 shows similar folding dynamics as that of SF206 (Fig. 4d) and SF217 (Supplementary Fig. S6), i.e., before the pseudoknot conformation forms stably, fast hopping between ssRNA and hairpin intermediate is observed for a few seconds as the force is dropped to 17.0 pN.

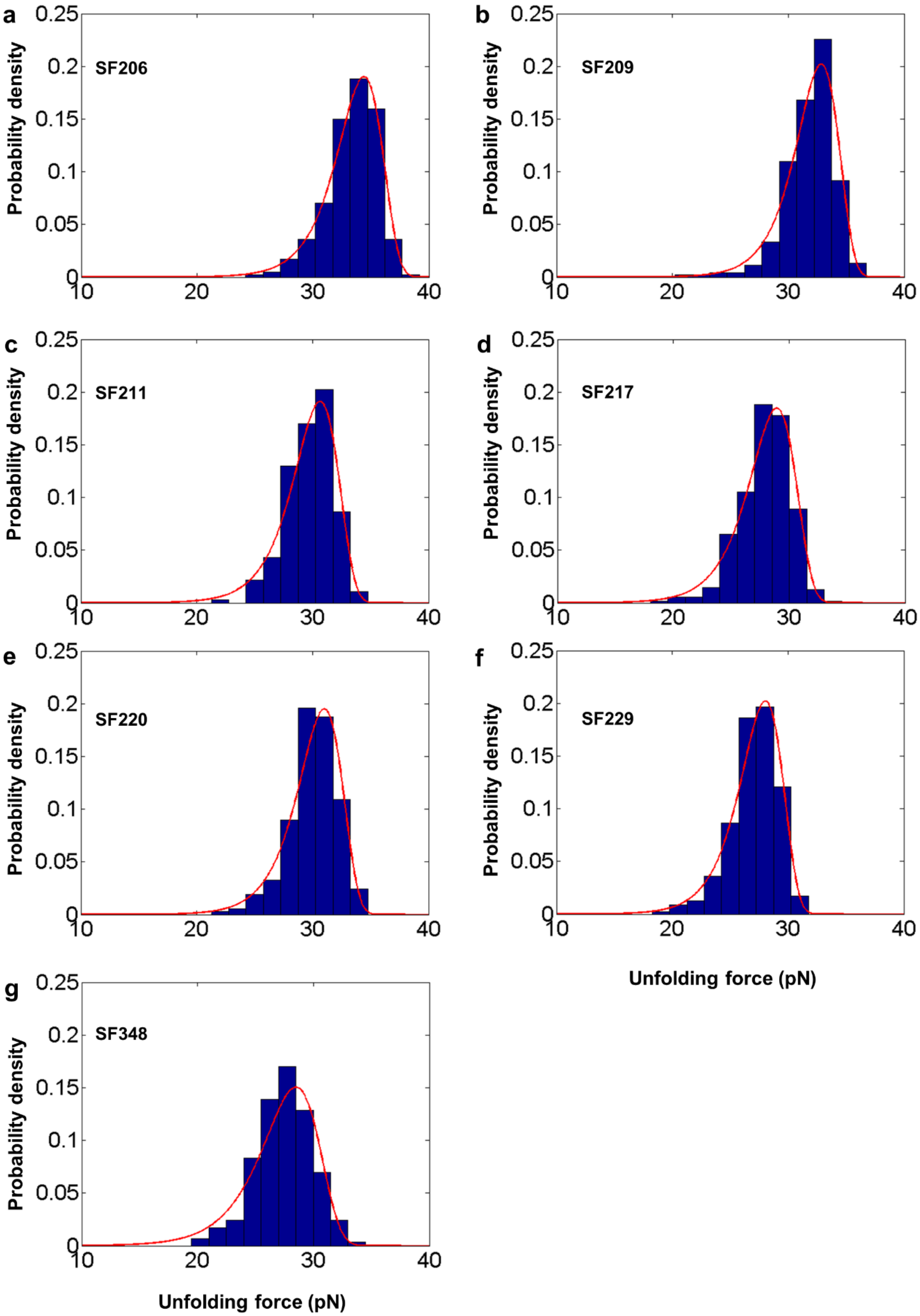

a
SF206
b
SF209
c
SF211
d
SF217
e
SF220
f
SF229
g
SF348
Probability density
Unfolding force (pN)
0.25
0.2
0.15
0.1
0.05
0
10
20
30
40

Figure S8. One-step rupture force distributions for pseudoknots from force-ramp experiment. The red lines are plotted using parameters from linear fitting in Supplementary Fig. S9.

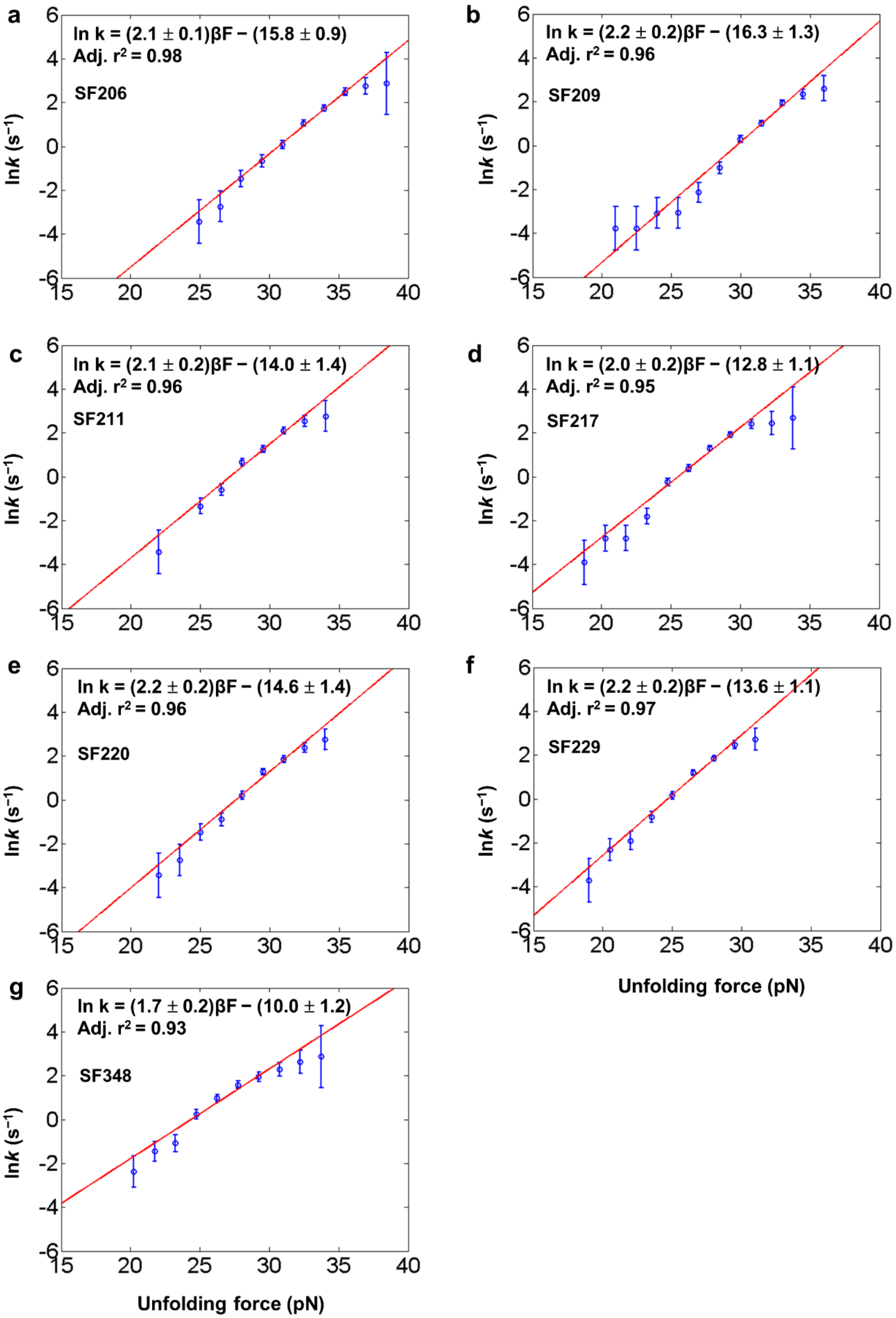
a
ln k = (2.1 ± 0.1)βF − (15.8 ± 0.9)
Adj. r² = 0.98
SF206
b
ln k = (2.2 ± 0.2)βF − (16.3 ± 1.3)
Adj. r² = 0.96
SF209
c
ln k = (2.1 ± 0.2)βF − (14.0 ± 1.4)
Adj. r² = 0.96
SF211
d
ln k = (2.0 ± 0.2)βF − (12.8 ± 1.1)
Adj. r² = 0.95
SF217
e
ln k = (2.2 ± 0.2)βF − (14.6 ± 1.4)
Adj. r² = 0.96
SF220
f
ln k = (2.2 ± 0.2)βF − (13.6 ± 1.1)
Adj. r² = 0.97
SF229
g
ln k = (1.7 ± 0.2)βF − (10.0 ± 1.2)
Adj. r² = 0.93
SF348
lnk (s⁻¹)
Unfolding force (pN)

Figure S9. Force-dependent unfolding rates extracted from rupture force distributions obtained from force-ramp experiment using Dudko's method.[1] Errors for the unfolding rates are calculated based on Poisson noise. The Bell model was applied to extract the unfolding rates at zero force or other forces (Fig. 7 and Supplementary Fig. S10) and unfolding transition state positions. Errors of ln$k$ are reported as standard errors.

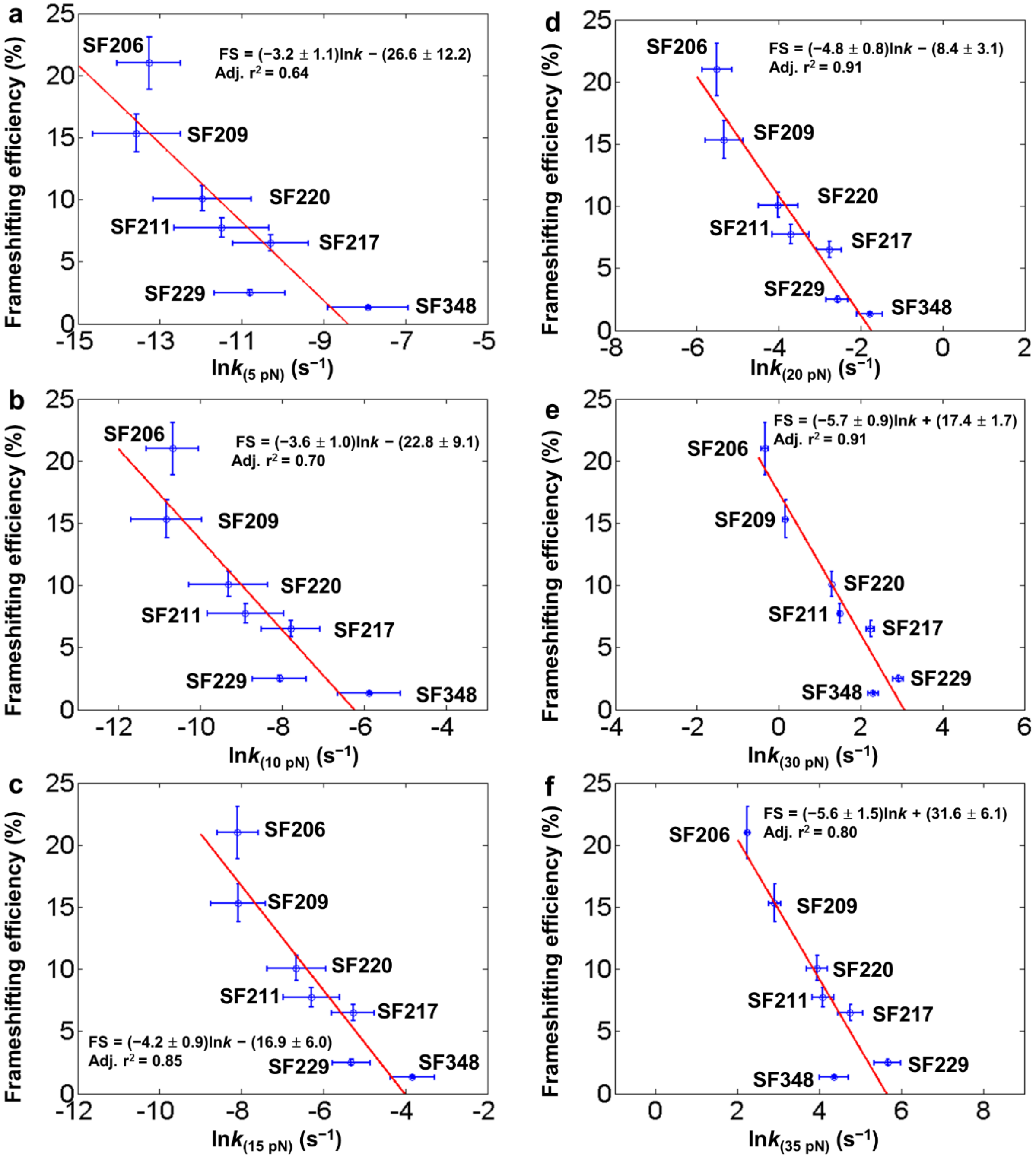

Figure S10. Previously measured *in vitro* frameshifting efficiency[28] versus unfolding rate, ln*k*, at varied forces obtained from force-ramp experiment. The plots for 0, 25, and 40 pN are shown in Fig. 7. Linear fitting was applied for each plot. Error bars of frameshifting efficiency represent standard deviations. Errors of ln*k* are reported as standard errors.

References

1. Dudko, O.K., Hummer, G. & Szabo, A. Theory, analysis, and interpretation of single-molecule force spectroscopy experiments. *Proc Natl Acad Sci USA* **105**, 15755-60 (2008).
2. Wu, Y.J., Wu, C.H., Yeh, A.Y. & Wen, J.D. Folding a stable RNA pseudoknot through rearrangement of two hairpin structures. *Nucleic Acids Res* **42**, 4505-15 (2014).
3. Bell, G.I. Models for the specific adhesion of cells to cells. *Science* **200**, 618-27 (1978).
4. Wen, J.D. et al. Force unfolding kinetics of RNA using optical tweezers. I. Effects of experimental variables on measured results. *Biophysical J* **92**, 2996-3009 (2007).
5. Bustamante, C., Marko, J.F., Siggia, E.D. & Smith, S. Entropic elasticity of lambda-phage DNA. *Science* **265**, 1599-600 (1994).
6. Neupane, K., Yu, H., Foster, D.A., Wang, F. & Woodside, M.T. Single-molecule force spectroscopy of the add adenine riboswitch relates folding to regulatory mechanism. *Nucleic Acids Res* **39**, 7677-87 (2011).
7. Liphardt, J., Onoa, B., Smith, S.B., Tinoco, I., Jr. & Bustamante, C. Reversible unfolding of single RNA molecules by mechanical force. *Science* **292**, 733-7 (2001).
8. Wang, M.D., Yin, H., Landick, R., Gelles, J. & Block, S.M. Stretching DNA with optical tweezers. *Biophys J* **72**, 1335-46 (1997).
9. Manosas, M. & Ritort, F. Thermodynamic and kinetic aspects of RNA pulling experiments. *Biophys J* **88**, 3224-3242 (2005).
10. Seol, Y., Skinner, G.M. & Visscher, K. Elastic properties of a single-stranded charged homopolymeric ribonucleotide. *Phys Rev Lett* **93**, 118102 (2004).
11. Michiels, P.J. et al. Solution structure of the pseudoknot of SRV-1 RNA, involved in ribosomal frameshifting. *J Mol Biol* **310**, 1109-23 (2001).
12. Hukushima, K. & Nemoto, K. Exchange Monte Carlo method and application to spin glass simulations. *J Phys Soc Jpn* **65**, 1604-1608 (1996).
13. Okabe, T., Kawata, M., Okamoto, Y. & Mikami, M. Replica-exchange Monte Carlo method for the isobaric-isothermal ensemble. *Chem Phys Lett* **335**, 435-439 (2001).
14. Hornak, V. et al. Comparison of multiple amber force fields and development of improved protein backbone parameters. *Proteins* **65**, 712-725 (2006).
15. Darden, T., York, D. & Pedersen, L. Particle Mesh Ewald - an N.Log(N) Method for Ewald Sums in Large Systems. *J Chem Phys* **98**, 10089-10092 (1993).
16. Bussi, G., Donadio, D. & Parrinello, M. Canonical sampling through velocity rescaling. *J Chem Phys* **126**, 014101 (2007).
17. Parrinello, M. & Rahman, A. Crystal-Structure and Pair Potentials - a Molecular-Dynamics Study. *Phys Rev Lett* **45**, 1196-1199 (1980).
18. Parrinello, M. & Rahman, A. Polymorphic Transitions in Single-Crystals - a New Molecular-Dynamics Method. *J Appl Phys* **52**, 7182-7190 (1981).
19. Patriksson, A. & van der Spoel, D. A temperature predictor for parallel tempering simulations. *Phys Chem Chem Phys* **10**, 2073-2077 (2008).
20. Grubmuller, H., Heymann, B. & Tavan, P. Ligand binding: Molecular mechanics calculation of the streptavidin biotin rupture force. *Science* **271**, 997-999 (1996).
21. Hummer, G. & Szabo, A. Free energy reconstruction from nonequilibrium single-molecule pulling experiments. *Proc Natl Acad Sci USA* **98**, 3658-3661 (2001).
22. White, K.H., Orzechowski, M., Fourmy, D. & Visscher, K. Mechanical unfolding of the beet western yellow virus -1 frameshift signal. *J Am Chem Soc* **133**, 9775-82 (2011).

23. Hess, B., Kutzner, C., van der Spoel, D. & Lindahl, E. GROMACS 4: Algorithms for Highly Efficient, Load-Balanced, and Scalable Molecular Simulation. *J Chem Theory Comput* **4**, 435-47 (2008).
24. Van Der Spoel, D. et al. GROMACS: fast, flexible, and free. *J Comput Chem* **26**, 1701-18 (2005).
25. Jorgensen, W.L., Chandrasekhar, J., Madura, J.D., Impey, R.W. & Klein, M.L. Comparison of Simple Potential Functions for Simulating Liquid Water. *J Chem Phys* **79**, 926-935 (1983).
26. Hess, B., Bekker, H., Berendsen, H.J.C. & Fraaije, J.G.E.M. LINCS: A linear constraint solver for molecular simulations. *J Comput Chem* **18**, 1463-1472 (1997).
27. Bergues-Pupo, A.E., Arias-Gonzalez, J.R., Moron, M.C., Fiasconaro, A. & Falo, F. Role of the central cations in the mechanical unfolding of DNA and RNA G-quadruplexes. *Nucleic Acids Res* **43**, 7638-47 (2015).
28. Olsthoorn, R.C., Reumerman, R., Hilbers, C.W., Pleij, C.W. & Heus, H.A. Functional analysis of the SRV-1 RNA frameshifting pseudoknot. *Nucleic Acids Res* **38**, 7665-72 (2010).
29. Garai, A., Zhang, Y. & Dudko, O.K. Conformational dynamics through an intermediate. *J Chem Phys* **140**, 135101 (2014).
